# Ribosomal History Reveals Origins of Modern Protein Synthesis

**DOI:** 10.1371/journal.pone.0032776

**Published:** 2012-03-12

**Authors:** Ajith Harish, Gustavo Caetano-Anollés

**Affiliations:** Evolutionary Bioinformatics Laboratory, Department of Crop Sciences, University of Illinois, Urbana-Champaign, Illinois, United States of America; University of California Santa Barbara, United States of America

## Abstract

The origin and evolution of the ribosome is central to our understanding of the cellular world. Most hypotheses posit that the ribosome originated in the peptidyl transferase center of the large ribosomal subunit. However, these proposals do not link protein synthesis to RNA recognition and do not use a phylogenetic comparative framework to study ribosomal evolution. Here we infer evolution of the structural components of the ribosome. Phylogenetic methods widely used in morphometrics are applied directly to RNA structures of thousands of molecules and to a census of protein structures in hundreds of genomes. We find that components of the small subunit involved in ribosomal processivity evolved earlier than the catalytic peptidyl transferase center responsible for protein synthesis. Remarkably, subunit RNA and proteins coevolved, starting with interactions between the oldest proteins (S12 and S17) and the oldest substructure (the ribosomal ratchet) in the small subunit and ending with the rise of a modern multi-subunit ribosome. Ancestral ribonucleoprotein components show similarities to *in vitro* evolved RNA replicase ribozymes and protein structures in extant replication machinery. Our study therefore provides important clues about the chicken-or-egg dilemma associated with the central dogma of molecular biology by showing that ribosomal history is driven by the gradual structural accretion of protein and RNA structures. Most importantly, results suggest that functionally important and conserved regions of the ribosome were recruited and could be relics of an ancient ribonucleoprotein world.

## Introduction

Translation is a complex and highly coordinated process of protein biosynthesis that is mediated by a universal ribonucleoprotein (RNP) complex, the ribosome. Ribosomes are composed of two major subunits [1]. The small subunit (SSU) consists of one ribosomal RNA (rRNA) molecule and more than 20 ribosomal proteins (r-proteins) depending on the species. The large subunit (LSU) consists of 2–3 rRNA and more than 50 r-proteins. Translation begins when the two subunits associate by establishment of intersubunit bridges [2]. The SSU mediates the interactions between messenger RNA (mRNA) and transfer RNAs (tRNAs) to decode genetic information and the LSU catalyses peptide bond synthesis [3]. The r-proteins generally occupy peripheral regions but have extended tails that penetrate into the functional core. While advances in structural studies showed the extensive mediation by RNA [1], it was recognized very early that both r-proteins and rRNA are required for efficient ribosomal functioning [4]. In addition to their role in ribosomal assembly and stability, r-proteins contribute significantly to all stages of translation [5]. In fact, recent biochemical and structural studies have shown that r-proteins stabilize and facilitate binding of tRNA and are determinants of the rate of peptidyl transfer [6], [7].

Many theories attempt to explain the emergence of the ribosome, including the idea that a simple primitive ribosome that passively facilitated translation [8], [9] refined its speed and accuracy with time [10]. Ribosome evolution is also intricately linked to evolution of tRNA and the genetic code. Several theories posit the triplet genetic code originated before translation and had functions distinct from extant molecules [11], [12]. While specific models vary, most theories propose that translation was a functional takeover of a primitive RNA-based replication apparatus. Although plausible, these theories have been highly speculative.

**Figure 1:**
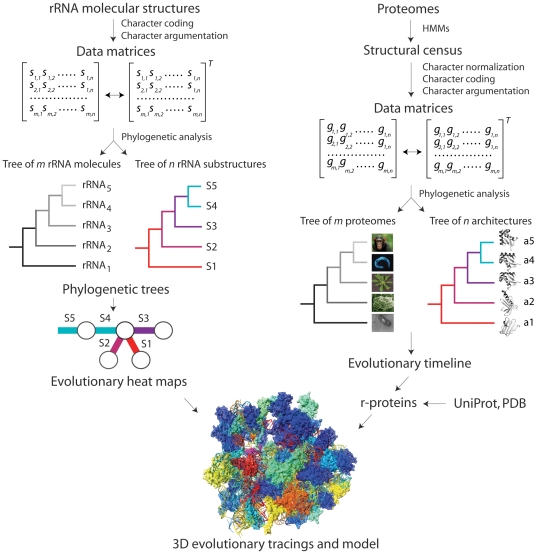
Experimental strategy. The flow diagram in the left describes the phylogenetic reconstruction of trees of rRNA molecules and substructures. The structures of rRNA molecules were first decomposed into substructures, including helical stem tracts and unpaired regions. Structural features of these substructures (e.g., length) were coded as phylogenetic characters and assigned character states according to an evolutionary model that polarizes character transformation towards an increase in molecular order (character argumentation). Coded characters (s) are arranged in data matrices, which can be transposed for cladistic analyses. Phylogenetic analysis using MP methods generate rooted phylogenetic trees of either molecules or substructures. Only trees of substructures are presented in this study. The flow diagram in the right shows the reconstruction of trees of proteomes and trees of protein domain structures. A census of domain structures in proteomes of hundreds of completely sequenced organisms is used to compose a data matrix and its transposed matrix, which are then used to build phylogenomic trees describing the evolution of individual protein structures and entire molecular repertoires, respectively. Elements of the matrix (g) represent genomic abundances of architectures (at FSF level of hierarchical classification of structure) in proteomes. Trees of proteomes will be described elsewhere, but are largely congruent with traditional classification. Embedded in the tree of rRNA substructures and tree of protein domains are timelines that assign age to molecular structures. These ages can be “painted” onto 2D or 3D structural models of the ribosome, generating evolutionary heat maps. Evolutionary information from RNA and protein structures is finally combined to generate a model of structural evolution.

Here we infer the origin and evolution of the ribosomal ensemble from phylogenetic methods applied directly to the structure of RNA [13], [14] or from a census of protein structures in proteomes [15]. The general approach we use (Figure 1) has been employed in a number of important applications, mines information in extant molecules, and generates rooted phylogenetic trees that embed structure and function directly into phylogenetic analysis (Text S1). Trees generated from an analysis of the structures of thousands of RNA molecules and from a census of protein domain structures in hundreds of genomes show that the structure of rRNA evolved gradually in conjunction with r-proteins. It also reveals that universally conserved, functionally important core components at the interface of SSU and LSU are primordial. We also present evidence for similarity of this core to *in vitro* evolved ribozymes and show that modern protein synthesis likely evolved from recruitment of related preexisting functions in primordial molecules.

## Results and Discussion

### rRNA History Reveals that an Ancestral Processivity Core Precedes the Emergence of the Peptidyl Transferase Center (PTC)

Intuitively, a large and complex molecular ensemble such as the ribosome must evolve through a stepwise process in which structural components are gradually added to the expanding molecules. This makes the age of these components necessarily diverse. Stimulated by the discovery of symmetry in the region that hosts the PTC and an origin that embeds a structural duplication [16], tertiary structure has been used to make inferences and simulate the evolution of LSU rRNA. These studies assume that helical-stack interactions recapitulate molecular growth [17] and structures grow in concentric shells from an ancient core that embeds the PTC [18], [19]. However, they do not employ a systematic comparative or phylogenetic framework and are limited to LSU rRNA in available crystal structures. In contrast, here we infer the history of the complete RNP ensemble using phylogenetic methods that employ standard cladistics principles widely used for example in the analysis of morphological characteristics of organisms. Shared-derived features of structure defined by crystallography and comparative sequence analysis are treated as phylogenetic characters and used to build structural phylogenies (Figure 1). We note that the historical statements we present are necessarily derived from ribosomal structures that exist today and not from those that were lost or are hypothetical.

**Figure 2:**
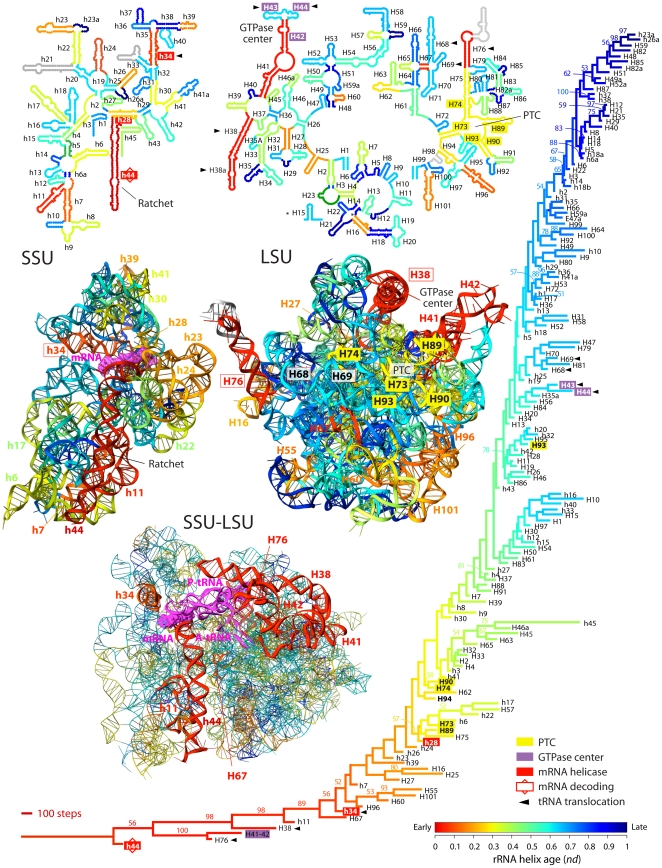
Evolution of rRNA structure. A strict consensus of 6 most-parsimonious trees (33,876 steps; CI = 0.168615, RI = 0.710934; HI = 0.831385; g_1_ = −1.425648) retained after a heuristic search with TBR branch swapping and simple addition sequence is colored according to relative age (*nd*) of extant (labeled taxa) or evolving (nodes) helical elements of structure. A total of 92 informative characters representing the structure of SSU and LSU rRNA in 93 organisms from the three superkingdoms were combined and analyzed. Bootstrap support (BS) values >50% are shown for individual nodes. Top and middle panels show evolutionary heat maps of *Thermus thermophillus* rRNA SSU and LSU rRNA secondary and crystal (2WDK and 2WDL) structures, respectively, with helices colored according to their age (*nd*). The lowest panel shows a primordial processivity core highlighted within the 70S ribosomal ensemble. Functional centers are highlighted in tree and heat maps.

**Figure 3:**
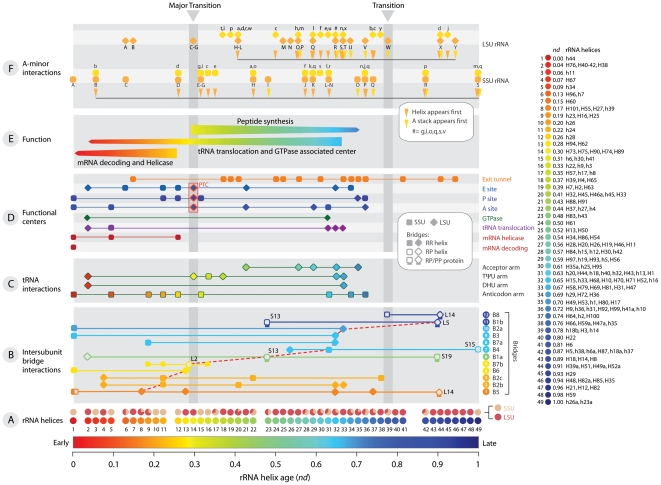
Timeline of development of the functional centers of the ribosome. **A**, The relative age (*nd*) of different rRNA helices (colored circles) increases from left to right and SSU and LSU functional elements are indicated with squares and rhomboids, respectively. Pie charts below each time point show the percentage of SSU and LSU helices appearing at that time, and the two periods of evolutionary transition are shaded. **B**, Timeline of structures in bridges. The age of bridge interactions is assigned as the age of first acceptor element of the donor-acceptor pair forming the bridge (red lines). **C**, Timeline of helices that interact with the different arms of tRNA. **D**, Timelines of helices that form the functional centers of the ribosome. The PTC is highlighted with a red box. **E**, History of functions. The width of the arrows portrays the increase of elements forming the center and time taken for its development. **F**, Timeline of A-minor interactions in SSU and LSU rRNA. Names with capital letters indicate the donor and in small case indicate the acceptor of the A-minor interaction.

Phylogenies of rRNA structural elements rooted by polarizing character change (from ancestral to derived) provide a chronology of accretion of substructures in molecules (Text S1). Hence, the tree in itself becomes a model of structural evolution. We reconstructed a universal tree of rRNA helical elements that are present in all three superkingdoms of life (Figure 2). Trees describing the separate evolution of helices in SSU or LSU rRNA built from structural data in ∼20,000 rRNA molecules were largely congruent and corroborate rRNA history (Figure S1). These structural trees are supported by three fundamental and well supported assumptions: (i) rRNA can be considered a 3-dimensional (3D) arrangement of helices [20], (ii) topological constraints of secondary structure greatly define global RNA structure [21], and (iii) rRNA can be decomposed into helices for evolutionary study [13], [17]. The number of internal nodes defining branch splits from the root to each leaf of the tree increases monotonically with time. We therefore calculated the relative age of each rRNA helix as a *node distance* (*nd*), the relative number of nodes along branches of the trees (Table S1). These relative ages were used to color secondary and 3D structural representations of the ribosome (evolutionary heat maps) (Figure 2) and to build timelines of accretion of components of the ribosome and their associated functions (Figure 3).

**Figure 4:**
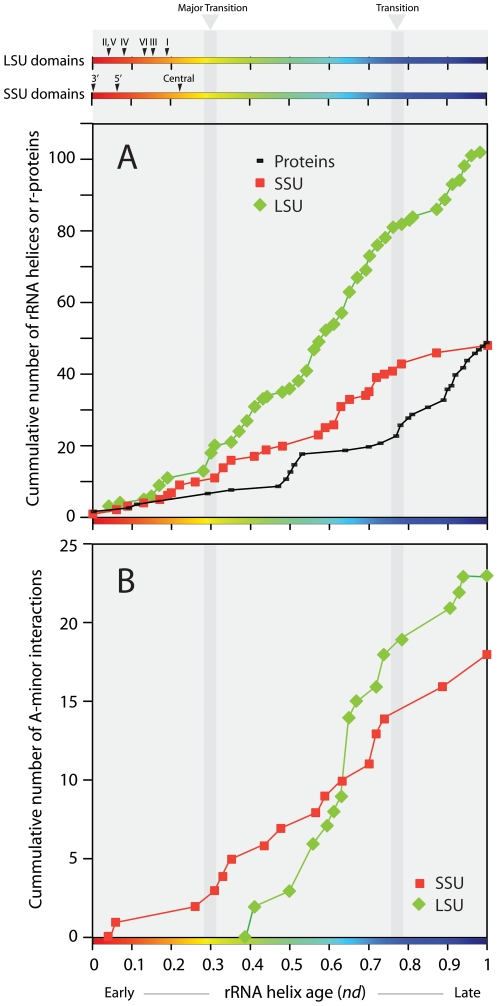
Evolutionary accretion of molecular structures and establishment of A-minor interactions. **A.** Cumulative plots describing ribosomal accretion of rRNA helices and r-proteins in the evolutionary timeline. Timelines at the top show the first appearance of individual structural-domains in rRNA subunits. Periods of evolutionary transition are shaded in grey. Note the rapid increase of structural complexity after the first transition, where processivity and peptide synthesis came together. **B.** Accumulation of A-minor interactions associated with individual rRNA subunits in ribosomal history. Plots describe the cumulative number of A-minor interactions as function of ribosomal age, as interactions accumulate in the evolutionary timeline of rRNA structure. The rapid increase in the number of A-minor interactions after the first transition, where processivity and peptide synthesis came together.

Phylogenetic trees show the penultimate helical stem h44 in the SSU rRNA is the oldest (Figure 2). This helix is the main component of the functional relay that links processes in the SSU decoding site with LSU-centered processes such as peptide bond formation and the release of elongation factors, thus modulating intersubunit interactions [22]. The timeline of accretion of the helical segments of the molecular ensemble reveals the concurrent structural diversification of the two major subunits (Figure 3A) and a proportional increase in subunit size at *nd*>0.3 (Figure 4). It also uncovers the functional origins of the ribosome, showcasing the early emergence and coordinated evolution of functionally important regions for ribosomal processivity in SSU rRNA responsible for mRNA decoding, tRNA translocation and mRNA helicase activities (Figure 3D and E). Their origin (*nd* = 0.0–0.3) precedes LSU substructures that make up the PTC, most of which appear together at *nd*∼0.3 (yellow helices H73–H75, H89, and H90 in Figure 2; Figure 3E). Remarkably, the rapid and coordinated appearance of PTC substructures in the trees (especially the H74 and H90 molecular speciation) (Figure 2) supports a possible duplication event responsible for the emergence of the PTC [16]. Driven by elongation factor G (EF-G), the ancient processivity core performs the mechanically complex function of ratcheting the subunits relative to each other and maintaining the reading frame and accuracy of translation. In contrast, the peptide bond synthesis activity of the more derived PTC is simpler (depends solely on the proximity and orientation of tRNA substrates [23]) and requires crucial contacts with the primordial core (Text S2).

We emphasize that the functional core involves the two subunits and is older than other regions of the ribosomal complex. Functional cores of individual subunits are centrally located in the 3D arrangements of corresponding subunits. Patterns of accretion of helices in our model are also consistent with PTC-first models that focus solely on the LSU rRNA [17], [18], [19]. A detailed comparison of molecular accretion (Figure S2) shows that models of LSU rRNA evolution can be reconciled (Text S2). We note however the benefits of a chronology of helices in both SSU and LSU rRNA, especially when coupled with a chronology of interacting r-proteins.

### Intersubunit Bridge History Indicates Early Independent Evolution of Subunits

The two major ribosomal subunits associate and communicate through intersubunit bridges and tRNAs in an interface that is almost devoid of proteins [2]. Since the intersubunit bridge interactions hold the ribosomal complex together we mapped these interactions to estimate when core ribosomal functions acted in concert. Figure 3B and Table S2 show the chronology of intersubunit bridge establishment. Bridge B5 is the oldest, first established between h44 and H27 (*nd* = 0.17). This initial bridge contact was followed by the appearance of h24-mediated contacts in bridges B2b and B2c (*nd* = 0.22). These first three bridges involve some of the oldest SSU and LSU helices (h44, h24, H67 and H27). Bridges B6 and B7b follow, preceding the formation of the PTC (*nd* = 0.28–0.29). They also involve h44 and h24, but establish contacts with an ancient r-protein, L2. Bridge B1a was then established (*nd* = 0.48) and was followed by the relatively quick appearance of bridges B4, B7a, B3, and B2a (*nd* = 0.63–0.67). Finally, B1b and B8 appear quite late in rRNA evolution (*nd* = 0.91). This progression of bridge interactions (red dotted line, Figure 3B) corresponds to the gradual accretion of ribosomal substructures. Bridges B5, B2b, B7a, B3 and B2a form the functional core of intersubunit contacts. Mutations in any of these contacts impair subunit association and translational fidelity [24]. Interestingly, about one half of this functional core (B5, B2b) and roughly one half of all helices involved in bridge contacts originate concurrently with the processivity center of SSU, while the other half of the functional core (B7a, B3, B2a) and remaining bridges originate after establishment of the PTC. The history of functions and interactions therefore suggests the two subunits functioned at first independently and that a ‘major transition’ in evolution of translation at *nd*∼0.30 brought the two subunits together into a modern protein biosynthetic ensemble. This transition likely coincided with the evolution of the tRNA cloverleaf.

### Tertiary Interactions Increase after the First Major Transition

rRNA secondary structure is specified largely by base paring and is stabilized by divalent cations and r-proteins [25]. However, multiple RNA-RNA and RNA-protein tertiary interactions between secondary structure motifs, such as pseudodoknots, tetraloops and A-minor interactions, provide additional stability. A-minor interactions were first described in the crystal structure of the LSU rRNA and are usually formed by highly conserved sets of nucleotides [26]. In addition to stabilizing rRNA structure, A-minor interactions play roles in decoding of mRNA [20]. The extent to which A-minor interactions are involved in ribosome function has prompted the study of their role in evolution of the LSU rRNA. The study is based on the assumption that the acceptor-helices into which adenosine stacks are inserted evolved before donor-helices [17]. We mapped all known A-minor interactions in both the SSU and LSU rRNA (Figure 3F). Indeed, the majority of the helices evolved before their corresponding adenosine stacks. Interestingly, >90% of these interactions occur after the first major transition (Figure 4), starting just after the development of the PTC and peaking around the time of development of the GTPase associated center (see below). During the ratcheting motion of mRNA-tRNA translocation in the elongation cycle, very large conformational changes are required [27]. We propose that A-minor and other tertiary interactions evolved to stabilize and maintain the ribosome structure during elongation, leading to increased ribosomal processivity. Scarcity of A-minor interactions before the major transition implies that the early proto-ribosome structure was mostly stabilized by r-proteins or their precursors. Although other RNA tertiary interactions could have played a role, it is less likely since they are not as abundant as A-minor interactions and they generally involve proteins [28]. It is also possible that the fewer helical structures of the proto-ribosome may have not needed tertiary interactions to be stable.

### tRNA is at the Center of Ribosomal Evolution

The proposed major transition corresponds not only to the rapid deployment of the PTC and bridges that link subunits but also to interactions with a full tRNA molecule in the A, P and E sites of the PTC (Figure 3C). tRNAs have two structurally and functionally independent halves with independent evolutionary origins [29], [30]. Each half interacts almost exclusively with one of the two ribosomal subunits [31], the ancient top half (composed of acceptor and TΨC arms) with the LSU and the derived bottom half (anticodon and dihydrouridine arms) with the SSU. Indeed, the timeline of tRNA interactions (Figure 3C) shows that among the known tRNA-rRNA interactions occurring before the major transition, many involved the ancient SSU helices and the relatively recent anticodon arm. After the transition, most tRNA-rRNA contacts involved newer LSU helices and the older half of the tRNA molecule. Establishment of crucial TΨC arm and SSU contacts (*nd* = 0.30–0.37) follow the emergence of the PTC (*nd* = 0.30) and makes this tRNA arm the only region capable of interacting with the two subunits. Contacts with the acceptor arm of tRNA necessary for peptidyl transfer, fidelity, and all steps of translation occurred later on (*nd* = 0.44–0.7). These remarkable patterns suggest that subunit interactions with a full modern cloverleaf tRNA structure were recruited for translation after the major transition and that the ribosome was built around tRNA or tRNA-like structures (Text S3).

### Structural Phylogenomics Reveals the Ribosome is an Ancient Coevolving RNP Complex

**Figure 5:**
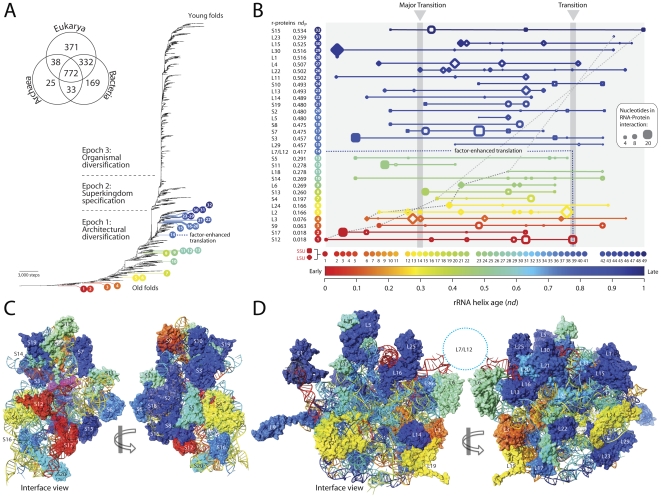
Relative age of r-proteins and their interaction with rRNA helices. **A**, Backbone of universal tree describing the evolution of 1,730 FSF domain structures from 749 genomes (541,383 steps; CI = 0.028, RI = 0.783; g1 = −0.111). The Venn diagram shows occurrence of FSFs in the three superkingdoms. **B**, rRNA helices establishing contacts with universal r-proteins. The relative age of the rRNA helices (*nd*) increases from left to right and r-proteins are ordered by age (from bottom to top) with corresponding *nd*_P_ value. The number of nucleotides at each time point involved in RNA-protein interactions is proportional to the size of squares (SSU) and rhomboids (LSU). r-proteins contacts are colored according to the age of the helix that makes the most ancient contact or is inferred from Figure S2. **C**, Evolutionary heat map of SSU r-proteins. **D**, Evolutionary heat map of LSU r-proteins. The 3D structures show the relative age of the rRNA helices and the relative age of r-proteins interacting with them.

r-proteins associate tightly with the ribosome, are extremely ancient, and their structures provide a unique window into early protein evolution [32]. To determine their relative age we generated a phylogenomic tree that describes the evolution of protein domains at fold superfamily (FSF) level of structural complexity (Figure 5A). The tree of domain structure is rooted (Text S4), was generated from a global genomic structural census in 749 proteomes using established methodology, and provides a timeline of appearance of proteins in the protein world that has considerable predictive power [15], [33].

We tested the existence of coevolutionary patterns by studying the age of universal r-proteins (*nd*_P_) (Table S3) and the age of rRNA helices (*nd*) they bind to (Figure 5B; Table S4). Coevolution is here defined as change in RNA that responds to change in proteins, and vice versa. The concept therefore implies the concurrent existence of molecular components that are somehow interacting with each other. The advanced linear hidden Markov models (HMMs) of structural recognition that we use in our structural census can identify r-protein domains reliably in proteomes, even in the presence of structurally disordered regions that lodge deep in the ribosomal core (see Methods). We do not expect the existence of these unstructured tails will bias the genomic abundance of domains and affect relative age estimates. Similarly, we do not expect that unstructured (unpaired) regions of rRNA structural elements will affect tree reconstructions, the age of rRNA helices, or the conclusions of our study. Remarkably, the oldest r-proteins, S12 and S17 (*nd*_P_ = 0.018), interact with the oldest (h44) and second oldest (h11) SSU rRNA helices, and equally remarkably, the linear correlation between the age of the most ancient rRNA contact (derived form the analysis of RNA structure) and the age of r-proteins (obtained from the census of domains in proteins) continues unabated until *nd*∼0.35 and *nd*_P_∼0.2 (dashed lines, Figure 5B). The correlation [*nd*_P_ = −0.535 *nd*+0.009; R^2^ = 0.961; F = 221.3, *P*<0.0001] was striking during early ribosomal history (Figure S3) and strongly suggests both RNA and proteins co-evolve together as RNA-protein interactions form with newly developed regions of the ribosome. The pattern of congruence also defines a general tendency that links protein and RNA timelines and reveals that r-proteins were steadily recruited throughout ribosomal evolution (Figure 4). We note that the early proteins, S12 and S17, also interact with helices h3, h4, h9 and h22, which are relatively recently derived (*nd* = 0.33–0.44). Similarly, many proteins start to interact with newer rRNA regions as they develop. Proteins appearing after the major transition also interact with older regions of rRNA. This indicates that r-protein precursors were interacting with the proto-ribosome very early in evolution and new interactions were continually established as rRNA structure evolved by accretion of new substructures and as the size of r-proteins increased in evolution to match helix growth and accretion (Figure 5C and D; Figure S4). We also note that rRNA and r-proteins could have existed before they established interactions. However, the striking congruence of the relative ages of rRNA and r-proteins, and the correspondence of these ages to the positions of the interacting RNA-protein segments in the 3D molecular arrangement (older components at the core of the ribosomal complex followed by newer components toward the periphery) is unlikely to be a fortuitous coincidence. Instead it should be taken as evidence of coevolution from very early stages.

The very early peptide chains were most likely synthesized by primitive means, perhaps through autocatalysis and/or non-ribosomal peptide synthesis (NRPS) [34], [35], since modern ribosomal translation had not yet evolved. A detailed model of early origins of primordial polypeptides and translation that is based on phylogenomic data [36] suggests the origin of modern biochemistry is linked to cellular membranes, acylation of thioesters, and non-ribosomal ligation of peptides [37]. In fact, timelines of protein domain structures at fold family level of structural abstraction show the development of domain structures with two active sites (catalytic-editing) capable of a two-step (activation-acylation) catalytic process developed before r-proteins and the modern ribosome [36], [37]. These domain structures are present in modern acyl-CoA synthetases, aminoacyl-tRNA synthetases (AARS) and NPRS acylating domains [37]. The chemical properties of these domains enable the donation of a highly diverse set of amino acid moeities to a multiplicity of substrates, a property that remains associated with protein biosynthesis in NRPS assembly lines [35] and AARS homologs of NRPS modules [38]. This links ribosomal and non-ribosomal peptide synthesis.

Biochemical studies of ribosomes depleted of several r-proteins [39] and structural studies of the LSU that revealed absence of proteins in the PTC was taken as evidence that the ribosome was a ribozyme [40]. Thus r-proteins were attributed only auxiliary roles in ribosome function. However, new revelations about r-proteins and catalytic mechanism of the ribosome have raised doubts about these views [41]. Biochemical studies and higher resolution structures of intact ribosomes with tRNA have shown that r-protein L27 stabilizes P-site tRNA in the PTC [6] and L16 facilitates aminoacyl-tRNA binding to the A site in bacteria [7]. Mutations in these two proteins substantially reduce the rate of peptidyl transfer. Ribosomal catalysis is thus a property of the integrated RNP complex rather than that of a confined section of RNA functional groups in the catalytic center [41]. Both protein and RNA have crucial roles that cannot be substituted with one another. Our phylogenomic analyses now provide strong evidence in favor of tight interdependence of r-proteins and rRNA (Figure 5, Figure S4).

Random polypeptides of the size of small proteins can fold into 3D conformations in the absence of selection [42]. Early peptides were therefore structured and likely rearranged and helped stabilize RNA, enabling rRNA structural conformations otherwise impossible by simple RNA-RNA interactions [43]. These changes induced small improvements in translation speed and accuracy, which provided strong selective advantages to the cells that carried them. We propose complex ribosomal functionality emerged from the cooperative interaction of rRNA and r-proteins (or their precursors), which existed from the earliest stages of ribosome evolution. Thus far, *in vitro* peptidyl transferase activity catalyzed by protein-free rRNA derived from extant rRNA or ribozymes is not demonstrated [44]. Perhaps, the primordial cooperative property of the RNP complex explains why such attempts have failed.

### Phylogenomics Reveals Early Origins of r-proteins and a Factor-Mediated Second Transition in Ribosomal Evolution

The tree of domain structure shows that S12 and S17 (*nd*_P_ = 0.018) are not only the oldest r-proteins but they appear after crucial metabolic proteins at the onset of the protein world (Figure 5A), early during a period of ‘architectural diversification’ (Epoch 1) [33], [36]. A modern RNP translation core evolved soon after, concurrently with L3, L2 and L24 (*nd*_P_ = 0.05–0.2) but long before many other r-proteins (most of which appear together in a narrow time interval, *nd*_P_ = 0.40–0.53) and long before the rise of superkingdoms in a diversified world (Figure 5A). A ‘gap’ in discovery of new proteins at *nd*_P_ = 0.32–0.40 signals a fundamental revision of the protein biosynthetic machinery, after which protein innovation is significantly enhanced. This second major transition in ribosomal evolution coincides with the emergence of the L7/L12 protein complex at *nd*_P_ = 0.42 and is followed by rapid r-protein diversification (Figure 5B). The L7/L12 complex stimulates the GTPase activity of EF-G, a ribosomal factor that catalyzes elongation and is responsible for marked increases in the processivity of the ribosome (Text S4).

### The Ribosomal Core Shares Structural Features with *In Vitro* Evolved RNA Ligase and Polymerase Ribozymes

**Figure 6:**
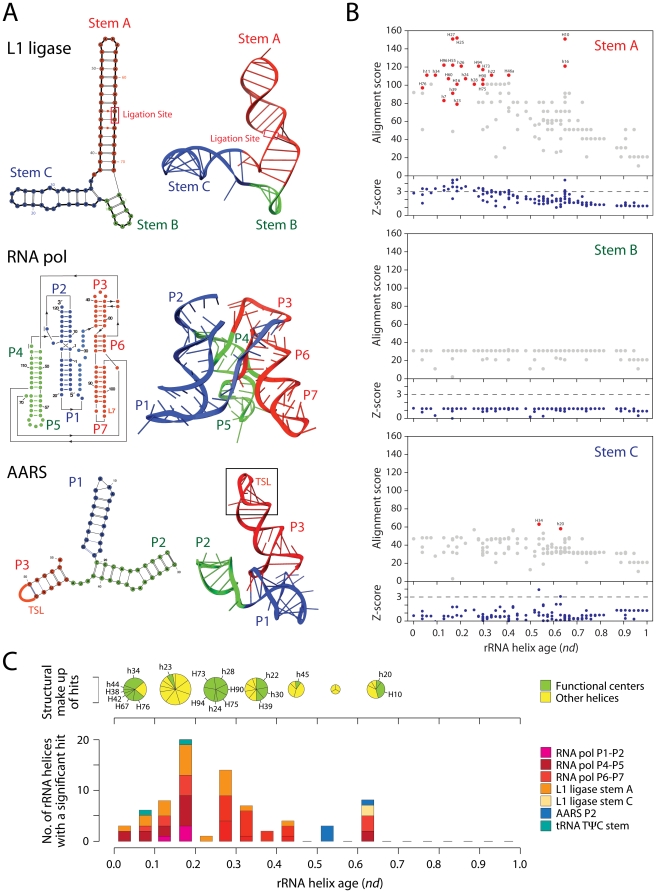
Similarity of ancestral rRNA structures to *in vitro* evolved ribozymes. **A**, Models of secondary and tertiary structure of L1 RNA ligase, RNA polymerase, and aminoacyl-tRNA synthetase (AARS) ribozymes. The long helix (stem A) of the 3-stemmed L1 RNA ligase molecule harbors the catalytic site and the junction of the three helical regions P1–P2, P4–P5 and P6–P7 at the center of the tripod-like RNA polymerase structure is the catalytic center. **B**, Alignment scores (top panels) and Z-score tests of statistical significance (bottom panels) for individual alignments of L1 ligase and rRNA helices of different age. Z-scores were derived from the alignment of 1,000 randomized sequences. Alignment scores of structures with Z-scores over 3 (horizontal dashed line) are significant at 0.01% confidence levels and are colored in red. **C**, Structural make up (pie charts) and frequency (bars) of rRNA helices of different age sharing structural features with the ribozymes. Only helices associated with functional centers (green pies) are labeled.

The absence of natural RNP polymerases, other than the ribosome, represents a gap in evolutionary continuity that precludes the phylogenetic analysis of ribosomal function. However, the biosynthesis of RNA (replication) and proteins (translation) share processive readings of RNA. Sequence and structure similarity searches between *in vitro* selected RNA replicase ribozymes and rRNA can uncover shifts in function during evolution (co-option) (Text S5). Substructures of L1 RNA ligase [45], RNA polymerase [46], and AARS [47] ribozymes (Figure 6A) and tRNA (used as a control) were aligned to substructures of hypothetical ancestral SSU and LSU rRNA (reconstructed directly from our trees; Figure S5). Figure 6B shows alignment statistics for substructures of the ligase ribozyme. Statistically significant similarity was detected preferentially between primordial rRNA helices (*nd*<0.3) and the catalytic helices of the ligase and polymerase ribozymes, but not with substructures of the AARS or tRNA molecules (Figure S6). Substructures sharing structural features with the ribozymes were part of functional centers (44%), most of which favor either nucleotide interactions or peptide bond formation, or had a structural supportive role (Figure 6C), and represented half of all functional substructures. Thus, it is likely that the ribosomal catalytic core had origins in processive substructures common to replication and translation and is a descendant of a primitive templating complex. These results in combination with biochemical evidence that shows that the processivity function of the PTC (peptide release) is more conserved and catalytically limiting than its central biosynthetic function (peptide bond synthesis) [48] provide crucial evidence in favor of functional recruitments. Since structural components of a proto-ribosome involved in tRNA, mRNA and intersubunit interactions are older than others, these results also support the replicative origin of tRNA [30], [49].

### Ancient OB-fold Proteins Linked to Replication were Recruited for Early Ribosomal Function

The oldest r-proteins are involved in different aspects of ribosomal processivity and extra-ribosomal functions linked to replication. For instance, S12 is involved in mRNA movement, tRNA translocation and forms the signal relay that communicates recognition of the correct tRNA to EF-Tu during decoding [50]. S17 is among the first proteins to stabilize 16S rRNA conformations nucleating the SSU assembly process [25]. Likewise, L3 maintains conformation of the PTC and is an allosteric switch modulating the binding of the elongation factors [51] and L2 in addition to being important for subunit association [52] binds to RNA polymerase to modulate transcription [53]. Remarkably, these primordial r-proteins share ancient structural designs, the OB-fold and the related SH3-like small β-barrel folds. Translation initiation factors, tRNA binding proteins including AARSs, DNA binding proteins like T7 DNA ligase, and telomere binding proteins share the same fold arrangement [54]. RNA binding and DNA binding proteins therefore have a common evolutionary origin, suggesting ancient r-proteins and homologs were originally part of primitive replication machinery, which diversified and was co-opted for modern translation. This ancient replicative function most likely involved processivity and biosynthetic activities that we believe remain hidden today in ribosome function (Figure S7).

### Gradual Evolution of Functional Novelty is an Expected Outcome

The origin of evolutionary novelty by recruitment or co-option of preexisting modules is well known in classic ‘evo-devo’ studies [55], [56] and is well studied in the case of RNA secondary structures [56], [57], [58]. In addition, it has been recognized that the genetic code links gene replication and expression, which are thus intricately related [10], [12]. Our results are consistent with the concept of evolutionary continuity where phenotypic transitions in evolving RNA structures are connected by a neutral network and small changes in sequence result in novel structures and functions [58], [59], [60]. Many important aspects of extant ribosome function corroborate our conclusions:

- Functional robustness of catalytic complexes depends on structural stability [61], [62], which is a result of ‘canalization’ of the structures towards increased resilience to perturbation [56]. Ribosomal robustness is in its processivity and in the accuracy of translating the genetic code [63], [64], [65]. Translational robustness thus affects organismal fitness [66]. The genetic code has evolved to be highly optimized and reflects coevolution of tRNA abundance and codon usage [12], [67] and is related to translational accuracy [68], which is ultimately constrained by aminoacyl-tRNA selection and mRNA-tRNA translocation [69].
- Kinetic studies have shown that codon-anticodon base paring initiates translation elongation and accelerates the induced-fit of substrate selection. Other template directed enzymes such as RNA and DNA polymerases use similar mechanisms [70], [71]. Moreover, the movement of tRNA in the 30S subunit limits the overall rate of translocation [72]. Thus, some degree of accuracy of tRNA selection is necessary for template-directed protein synthesis. This justifies our model of evolution of the modern ribosome centered on tRNA and SSU structural components. Accuracy of selection, rate of selection and direction of the tRNA-mRNA translocation is greatly enhanced by r-proteins and translation factors [73], [74] and supports our interpretation of very early RNA-protein cooperativity.
- Finally, evidence for an ancient tRNA-centered ribosomal replication apparatus can be found in many aspects of mRNA-tRNA translocation during translation. The accuracy of mRNA-tRNA translocation requires an aminoacyl-tRNA in the P-site [75], the SSU E-site is crucial in maintaining the reading frame [76] and secondary structure and tertiary interactions in rRNA have evolved for specific intersubunit communication that follows the deacylation of A-tRNA during translocation [77].

These aspects are consistent with the ‘triplicase’ model proposed for a primitive replication apparatus that could potentially be co-opted for translation [11], which agrees well with our evolutionary model.

### Conclusions

**Figure 7:**
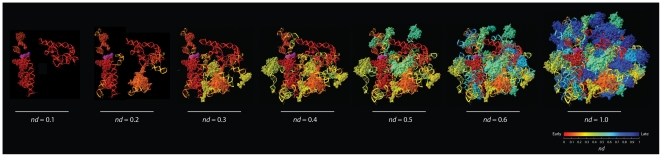
Model of ribosomal evolution. A chronological representation of the evolution of the ribosome shows that very early in ribosomal evolution (*nd*<0.3) rRNA helices interacted with r-proteins to form a processivity core that mediated nucleotide interactions, which later (*nd* = 0.3) served as center for coordinated and balanced RNP accretion leading to modern ribosomal function. The purple structure indicates extant mRNA, which is used as structural reference for location of primitive functional centers. We envision the primordial ribosome had replicative functions that likely involved RNA, so the mRNA molecule from the crystallographic model should be regarded as placeholder for the ancient coding molecule. rRNA is rendered as ribbon representation, mRNA and proteins as rendered as space-filling representations.

Although a primitive ribosome composed solely of RNA has been proposed [78], [79], it is unlikely that such a complex RNA machine could have existed. Instead, it is likely that multiple smaller RNP complexes with different functions integrated during evolution into a much more complex RNP ensemble. Arguments that support a peptide synthesis-first origin of translation are based on the premise that the triplet genetic code could not have evolved if it had no associated function [81]. However, origins of evolutionary novelty by ‘functional shifts’ induced by molecular recruitment are common and can explain modern ribosomal activities. In this study we provide phylogenetic evidence that explains the origin and emergence of the ribosome, and crucial evidence in support of primordial RNP machinery, which late in protein evolution gave rise to coded protein synthesis. The roles of ancient RNP components were not fixed (canalized) from the beginning and are probably still evolving. Our data is consistent with: (1) modern peptide synthesis arising as a secondary process that facilitated primitive processive readings of RNA; (2) the emergence of translation from simpler, separate processes, once these assembled around a primordial tRNA with coding capacity; and (3) the displacement and ultimate take-over of an initial templating complex by integration of separate component parts into modern catalytic machinery. We propose that the emergence of a complex RNP translation apparatus, summarized in the serial timeline of Figure 7, improved the production and quality of proteins. These proteins took over most functions in a cell in a fundamental revision of cellular machinery. Such revision had profound influence in the protein world, as revealed by punctuation in timelines describing the evolutionary mechanics of domain organization in proteins [80] and biphasic patterns in the evolution of domains [36]. We show however that RNA played a crucial role in the emerging ribosomal RNP complex from the start as r-proteins co-evolved tightly with rRNA structure and organized around tRNA in the emerging translation system. We contend that RNA may be better suited than proteins for certain dynamic functions that are facilitated by repeated building-breaking of base pairing interactions. These functions include recognition of tRNA substrates, subunit associations, and large-scale movements of tRNAs and subunits [82]. Alternatively, rRNA may be just a contingency of history.

## Methods

### rRNA Data

The sequences and structures of LSU and SSU rRNA were obtained from the European Ribosomal RNA database (ErRD) [83]. ErRD secondary structures inferred by comparative sequence analysis were downloaded in DCSE format from http://bioinformatics.psb.ugent.be/webtools/rRNA/ (September 2005), with secondary structure encoded in helix numbering lines for sets of alignments specific to molecules of superkingdoms; Archaea, Bacteria or Eukarya. Helix numbering lines identify the corresponding paired regions of each helix in the secondary structure. A total of ∼600 LSU rRNA and ∼20,000 SSU rRNA sequences were obtained, after excluding more than 200 partial sequences. We first selected data for analysis from an initial study of rRNA evolution that included 35 sequences sampled from all three organismal superkingdoms of life [13]. Since ErRD is heavily biased towards bacterial sequences, a balanced set of 93 rRNA sequences representing 31 representative molecules of species in each superkingdom were selected and used to build trees (Table S5). Results presented in this manuscript focus on this set, which encompasses all universal rRNA structural elements (substructures) and major thematic variations of secondary structure that exist in the molecules. Finally, all usable sequences were analyzed, including a set of 593 LSU rRNA and 19,184 SSU rRNA sequences. Because our study does not represent a systematic analysis to discriminate species, representative sampling is an appropriate strategy.

### Phylogenetic Analysis of rRNA Structure

Since there are no explicit phylogenetic models for the evolution of RNA structure we reconstructed the history of molecular substructures in RNA molecules with maximum parsimony (MP) (implemented in PAUP* [84]) using methods we described previously [13], [14]. Phylogenetic relationships are inferred on the basis of shared and derived characteristics in structure with standard cladistic principles. RNA secondary structures were first characterized using attributes that describe the overall ‘shape’ (geometry) of the molecules, i.e. the topology of the folded conformations [56], [59]. In this study, we treat RNA secondary structures as planar abstractions of 3D folds and we do not focus on other alternatives, such as attributes that describe thermodynamic stability using minimum Gibbs free energy increments or statistics that measure the stability and uniqueness of the molecules, which have been also used successfully in our analyses (e.g., [13], [14], [30]). The structures of molecules we analyzed were first decomposed into substructural components. Structural features of homologous substructures (e.g., length of stems) were then treated as linearly ordered and polarized multi-state phylogenetic characters. These characters are used to build data matrices for MP tree reconstruction. The reconstructed trees describe a finite molecular system in which the ‘leaves’ represent the individual structural components of the molecule (Text S1). Sun and Caetano-Anollés [30] in their Figure 2 describe an example run of character coding and analysis. Phylogenetic analysis requires three methodological steps (Figure 1):

#### (i) Character coding

Topographic correspondence is the main criterion for determining character homology. When analyzing molecular structures, structural elements (substructures) are defined and mapped in space in the context of the entire molecule (i.e., the relative position of substructures in the rRNA molecules are established) and are then tested to determine if they represent true homologies acquired from a common ancestor. In our study, structural features were coded as multistate characters by establishing the length and number of helical stems (S), hairpin loops (H), bulge and interior loops (B), and unpaired sequences (U). Character states are based on the length (number of bases or base pairs) of these S, H, B and U substructures. Note that unpaired nucleotides sometimes form unusual base pairings or non-covalent interactions that delimit high-order 3D motifs [85]. Motifs such as tetraloops, pseudoknots, and A-minor interactions stabilize tertiary and quaternary structures, but are not considered for phylogenetic analysis in the structural models of this study. Consequently, coding of characters coarse-grains higher order structure into a simple framework of non-interacting helical segments. In rRNA, analysis of crystal structures of individual rRNA molecules or the ribosomal ensemble corroborates this framework. Nearly all of rRNA is helical or approximately helical, and RNA structure can be effectively considered a 3D arrangement of helical elements [20]. While character coding relies on correct prediction of secondary structure, covariation-based comparative sequence analysis has been successful in predicting structures with accuracies of up to 96% [86]. Structural inaccuracies at secondary structure level were therefore assumed not to be severe and were tolerated as systematic error, provided structures result from the same comparative sequence study. The coding of rRNA was based on secondary structure models for the large and small subunits inferred by comparative sequence analysis from sequences deposited in ErRD [83]. The SSU rRNA model contains 50 universal helical stems and several stems specific to Eukarya. The LSU rRNA model contains 100 universal stems and several other stems specific to certain taxa. These models are robust and have been verified by crystallography [30]. Only helices present in all three superkingdoms were used for the analysis and were defined as molecular segments separated by either multibranched loops (multiloops) or pseudoknotted loops. Structural alignments listed characters describing rRNA structure in the 5′-to-3′ direction as it is read in the sequence, and for each sequence segment, in the order S, B, H, and U. Stem substructures (S) were defined by two complementary sequence segments and corresponding characters (named by an alphanumeric descriptor and its prime). Helices were named using ErRD nomenclature [83], [87] for character coding and tree reconstruction. SSU rRNA helices were numbered S1–S50 and LSU rRNA helices were named with A-I (corresponding to ErRD LSU rRNA domains) and a number (e.g. A3, helix 3 of domain A). This nomenclature was reconciled with the standard Brimacombe nomenclature system [88] used in the crystal structure of *Thermus thermophillus* ribosome [31] (*see* Table S1). In phylogenetic analysis, character states were limited by the maximum number accepted by the phylogenetic analysis program (usually 64 states; [84]) and were represented by the numbers *0–9*, case sensitive alphabets *A-Z* and *a-z*, and special characters *@* and *&*. Structural features with longer than 64 nucleotide lengths were given the maximum state (&), and if missing, the minimum state (0). An in-house software module, marten [89], was used to code characters from DCSE alignments and to generate executable files for PAUP*.

#### (ii) Character argumentation

Character attributes represent transformation pathways and hypotheses of relationship that are falsifiable and link character states to each other using basic evolutionary assumptions or axioms [90]. Phylogenetic analysis of RNA structure rests on a very simple model of change in which geometrical or statistical features of structure (e.g. length of structural elements; values of Shannon entropy of the base pairing probability matrix) increase or decrease in value and on the auxiliary assumption (hypothesis of polarization) that there is an evolutionary tendency towards conformational order. Molecules in solution express different degrees of freedom, usually in the form of translations and rotations (e.g. internal rotations around single bonds) or dynamic motions that define different molecular conformations. In RNA, degrees of freedom are notably constrained by the formation of hydrogen bond interactions responsible for base pairs. This interplay is highly frustrated. Statistical mechanic simulations have successfully modeled the formation of secondary structure in RNA and the impact of mutation on structural change [56], [57]. We based our polarization hypothesis in this model. Within the range of free energies accessible at a given temperature, an RNA molecule folds into an ensemble of possible conformations (shapes). This ‘plastic repertoire’ delimits the time the RNA spends in each conformation. Molecular functions impact the fitness of an organism and are usually linked to certain conformation within the plastic repertoire, which are selected during evolutionary change. The more time a molecule spends in favored conformations the greater the molecule's impact on the organism's fitness. During selection, sequence mutants optimize folding to fewer thermally accessible conformations, most of which resemble the target and are most stable, spending more time in them. Moreover, the numbers of conformations that are accessible to the mutants also decreases and fold to nearly the target. This ‘lock-in’ process of structural canalization is autocatalytic and defines a general evolutionary trend of RNA molecules towards uniqueness, greater stability, and modularity. We here use this trend as hypothesis of character polarization by treating character states corresponding to increased structural order as being ancestral (plesiomorphic). Although this is a falsifiable hypothesis, thermodynamic, molecular mechanic, and phylogenetic considerations provide considerable theoretical and experimental evidence to support the polarization trend. These arguments have been recently summarized [91] and some are here revisited: (a) *Thermodynamic arguments*. The thermodynamic theory of evolution [92], [93] develops general principles that are applicable to biological systems of all hierarchies, ranging from molecular ensembles to ecosystems [94]. According to this theory, biological systems are self-organizing and tend to increase the order and complexity of the system by dissipating the disorder to their surroundings. These thermodynamic principles generalized to account for non-equilibrium conditions have experimentally verified a molecular tendency towards order and stability driving biological change [95]. (b) *Molecular mechanic arguments*. A large body of theoretical evidence that maps the structural repertoire of evolving RNA sequences from energetic and kinetic perspectives confirms evolution enhances conformational order and diminishes conflicting molecular interactions [56], with some important predictions supported experimentally [58], [96]. Studies of extant and randomized RNA sequences have also shown these tendencies. Randomizations of mono- and dinucleotides in single-stranded nucleic acids have been used to assess the effects of composition and order of nucleotides in the stability of folded molecules, uncovering evolutionary processes acting at DNA and RNA levels [97]. In recent experiments, extant evolved RNA molecules encoding complex, functional structural folds were compared to oligonucleotides corresponding to randomized counterparts [98]. Unlike evolved molecules, arbitrary sequences were prone to having multiple competing conformations. In contrast to arbitrary proteins, which rarely fold into well-ordered structures [99], these arbitrary RNA sequences were however quite soluble and compact. They appeared delimited by physicochemical constraints such as nucleotide composition that were inferred in previous computational studies [96]. (c) *Phylogenetic arguments*. Tendencies towards structural order and the hypothesis for rooting of trees have been experimentally verified by phylogenetic congruence between trees generated from RNA sequence and those generated from structure [12], [13], [100], in addition to congruence between phylogenies generated from geometric and statistical characters [30], [99], [101]. Polarizing characters in the opposite direction resulted in trees that were less parsimonious and had topologies incompatible with conventional taxonomy. Phylogenetic analyses testing hypotheses of organismal origin derived from global trees of tRNA structures and constraint analysis [102] and phylogenies of proteomes derived from an analysis of protein structures in entire genomic complements [33] proved to be congruent. They provide further indirect support to our hypothesis of polarization. Interestingly, we found character state changes are considerable, for example, along the basal branches of trees of helical substructures and in several other places of the tree (Figure S8). This suggests that the ancestral placement of basal helices (e.g., h44) does not result from helices being longer or from a ‘long branch attraction’ artifact. It also shows that stability and frustration of substructures are indeed important and congruent factors shaping the structure of rRNA. Since many of these structural components are functionally important, the increased frequency of character state change could reflect the various adaptations that are unique to organisms in different environments involved in the regulation of the translation process.

#### (iii) Phylogenetic reconstruction

Phylogenetic trees describing the evolution of rRNA structural elements were finally built using MP in PAUP* v. 4.0-b10 [85]. In this study, we present trees describing the evolution of rRNA helical stems, since stems are responsible for 3D patterns of molecular accretion, which are mostly defined by base-pairing interaction. Results obtained using trees of other structural components (H, B and U) inform about evolution of unpaired segments of the rRNA molecules and will be described elsewhere. The ancstates command was invoked to define ancestral character states and polarity of character transformation. Trees were derived from heuristic searches using tree-bisection-reconnection (TBR) branch swapping and simple addition sequence. Phylogenetic reliability was tested by the nonparametric bootstrap method implemented using 5,000 pseudoreplicates. Character reconstruction exercises were performed with macclade [103]. Tree topologies were analyzed using N_bar and cherry counts, statistics that provide information about symmetry and processes of speciation in trees. N_bar is the number of internal nodes between the base and the tips of the tree [104] and the cherry count is the number of internal nodes that have only terminal leaves as children [105]. These statistical measures of imbalance were implemented in TreeStat v. 1.2 (http://tree.bio.ed.ac.uk/software/treestat/) for trees of RNA substructures, random trees generated from structural data using PAUP*, and trees that follow the uniform speciation (Yule) model generated using Mesquite v 2.75 [106]. Since our method produces rooted trees that are highly unbalanced and reject the Yule and random speciation models (Text S1), the relative age (ancestry) of the individual structural elements in the trees could be approximated by measuring the distance in nodes (*nd*) from the hypothetical ancestor (root) in a relative 0–1 time scale. *nd* counts the number of cladogenic events (nodes) along each and every one of the lineages of the tree starting with the first event (root) and ending at the leaves. We calculated *nd* values with a perl script that counted the number of internal nodes along a lineage from the root to a terminal node (a leaf) of a given rooted tree with the following equation: *nd_a_* = (# of internal nodes between nodes *r* and *a*)/(# of internal nodes between nodes *r* and *m*), where *a* is a target leaf node, *r* is a hypothetical root node, and *m* is a leaf node that has the largest possible number of internal nodes from node *r*. Consequently, the *nd* value of the most ancestral taxon (helix) is 0 while that of the most recent one is 1. We note that when speciation (in our case structural speciation) depends on an evolving ‘heritable’ trait (e.g. the accumulation of mutational changes in structural features of RNA) the resulting phylogenies are expected to be highly unbalanced [107]. Under such circumstances *nd* becomes a good proxy for time as long as diversification rates do not vary across lineages. We also note that simulations that incorporate statistical mechanic considerations have shown that changes in RNA structure are generally discontinuous, with mutation resulting in long periods of stasis (as molecules drift in neutral networks) followed by sudden adaptive progress induced by structural transformations [59].

### Structure Alignments between *In Vitro* Engineered Ribozymes and rRNA

To detect remote homologies between structural elements of rRNA and ribozyme doppelgängers we used the structure alignment software RNAforester [108]. RNAforester is designed for pairwise and multiple RNA secondary structure alignments and is capable of detecting similar structural motifs based solely on conserved structure, independent of position and sequence conservation. The alignment procedure is essentially an equivalent of the Smith-Waterman (SW) algorithm [109] but applicable to RNA structures. However, unlike the SW algorithm, the scoring scheme is dependent on edit distances instead of alignment distances and sequence contributions to the score are negligible. Note however that although scoring is solely based on structural similarity sequence information can be used to improve the alignments.

In order to simplify the structure comparison exercise and to minimize effects of sequence variation in the large number of the rRNA sequences used in the study, hypothetical SSU and LSU rRNA ancestor sequences and structures were reconstructed using the maximum likelihood methods implemented in PAUP*. We reasoned that a reconstructed model is better than a consensus model. The process of sequence and structure reconstruction is summarized in Figure S5. Phylogenetic trees of rRNA molecules describing the evolution of 102 SSU or LSU rRNA molecules (representing organisms in the three superkingdoms of life) were reconstructed using structural data as previously described. The corresponding DCSE sequence alignments were then converted to FASTA and NEXUS format with SeqVerter (GeneStudio Inc., Suwanee, GA, USA) for use with PAUP*. Ancestral sequences for the hypothetical ancestors at the root of the trees were determined by reconstructing character states of all internal nodes with the ‘describe trees’ function and maximum likelihood methods in PAUP*. The best-fit model of nucleotide substitution (GTR+I+G) was selected by AIC with jModeltest v 0.1.1 [110]. The reconstructed sequences were manually reconciled to the DCSE alignment to obtain an alignment based on the secondary structure of the rRNA. The structure was then manually encoded into the Vienna format for use with RNAforester. Similar reconstructions were obtained for tRNA (from an analysis of 571 sequences).

The structures of ribozyme doppelgängers (L1 ligase, RNA polymerase, and aminoacyl-tRNA synthetase ribozymes) and the reconstructed structures of SSU rRNA, LSU rRNA, and tRNA were further decomposed into individual helices as defined by secondary structures, crystal structures, and criteria outlined above. Decomposed rRNA structures matched helices used in phylogenetic analyses and preserved hairpin loops, internal loops and bulges of these evolutionary units of structure. Pairwise local alignments were performed with each rRNA helix and each ribozyme doppelganger helix. Alignment scores were compared to determine which alignments had the best matches. Scores for individual rRNA helices were then plotted as function of helix age (*nd*). To establish the statistical significance of these alignments a background model of the structures derived from randomized sequences of the doppelgängers and control tRNA were also aligned to the rRNA helices. A total of 1,000 randomized sequences that preserve the dinucleotide frequency and sequence composition were generated as previously described [111]. The secondary structures of the randomized sequences were inferred using RNAfold from the Vienna RNA Package v1.8.4 [112]. The obtained structures were aligned to the reconstructed rRNA helices with RNAforester and statistically significant alignments were determined using Z-score statistics. Z-scores are commonly used as a measure of statistical significance of alignments when expectation value (e-value) statistics are not available [113]. A threshold Z-score of 3.0 was used to determine if the similarity measures of alignment scores were statistically significant at 0.01 confidence levels.

### Phylogenomic Analysis of Protein Domain Structure and Ancestry of r-Proteins

The general scheme applied to the evolutionary study of rRNA structure has been applied to the evolutionary study of protein domain structures [15], [33]. The scheme is illustrated in Figure 1. We first conducted a census of genomic sequence in 749 organisms that have been completely sequenced (52 archaeal, 478 bacterial, and 219 eukaryal species) assigning protein structural domains at FSF level of structural complexity to protein sequences using linear HMMs of structural recognition in superfamily [114] and probability cutoffs *E* of 10^−4^. Domains were defined by SCOP version 1.73 [115], [116] and described using SCOP *concise classification strings* (*ccs*). *ccs* descriptors are widely used symbolic representations of domains within the hierarchy of structural classification (e.g., the P-loop hydrolase FSF is named c.37.1, where c represents the protein class, 37 the fold and 1 the FSF). Features that numerically characterize the genomic abundance of each FSF (*g*) were used as characters to build data matrices for phylogenetic analysis. *g* indicates the number of multiple occurrences of an FSF domain in a proteome. Empirically, *g* values range from 0 to thousands and resemble morphometric data with a large variance [116], [117]. Because existing phylogenetic programs can process only a limited number of phylogenetic character states, the space of *g* values in the matrix was reduced using a standard gap coding technique developed for cladistic analysis of morphometric data [118]. We used the following formula to transform the data,
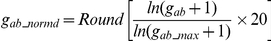
with *a* and *b* denoting an FSF and a proteome, respectively. *g_ab_* represents the *g* value of FSF *a* in proteome *b* and *g_ab_max_* indicates the maximum *g_ab_* value in all FSF in an individual proteome. This round function scales *g_ab_* to a 0–20 range and the 21 normalized *g* values represent character states and are encoded in nexus format as linearly ordered and polarized multistate phylogenetic characters using an alphanumeric set of numbers 0–9 and letters A-K that is compatible with PAUP*. Character states were polarized from ‘K’ to ‘0’ using the ancstates command in PAUP* based on two fundamental premises: (1) protein structure is far more conserved than sequence and carries considerable phylogenetic signal, especially at high levels of structural organization of this study (FSF), and (2) FSF that are successful and popular in nature are generally more ancestral, making ‘K’ the most ancient character state and ‘0’ the most recent. Details and support for character argumentation and absence of circularity in assumptions have been described and discussed previously [15], [33], [80], [119].

Universal phylogenetic trees of protein domain structure were built from the matrices using MP as the optimality criterion in PAUP* and rooted by the Lundberg method [85]. Because trees are large and the search of tree space is computationally hard, we used a combined parsimony ratchet (PR) and multiple iterative search approach to facilitate tree reconstruction and avoid the risk of optimal trees being trapped by sub-optimal regions of tree space [44], [80]. A recent review summarizes the general approach and the progression of census data and tree reconstruction in recent years [120]. Since trees are rooted and are highly unbalanced, we unfolded the relative age of protein domains directly for the phylogeny as a distance in nodes (*nd*_P_) from the hypothetical ancestral structure at the base of the tree in a relative 0–1 scale, essentially as we described for trees of rRNA structures. r-protein domains were mapped in trees of FSF domain structures and their corresponding *nd*_P_ values calculated to unfold the relative r-protein age. *nd*_P_ can be a good measure of age given a rooted tree since the semi-punctuated emergence of protein domains (i.e. taxa) is displayed by their ability to diverge (cladogenesis or molecular speciation) rather than by the amount of character state change that exists in branches of the tree (branch lengths) [117]. We note that while trees and timelines generated from abundance or occurrence of domains in genomes were not significantly different, phylogenetic analyses depend for example on the accuracy and balance of genomic databases (especially related to how representative they are of the biosphere), efficient and accurate assignment of structures to protein sequences, and methods of phylogenetic tree reconstruction. However, we do not expect that the effect of biases (e.g., faulty detection of FSFs with HMMs, over-representation of organisms in superkingdoms) will seriously affect the conclusions of this study (discussed in [15]).

In the dataset of universal r-proteins (Table S3), most proteins are made up of only one domain. In this case the age of the protein is the age of the domain. However, r-proteins L2, S3, S5, L11 and L10 are made up of two domains. In this case, the second domain added to the protein could be an ancient domain that was co-opted or it could be a new domain that was recruited to enhance the old function. To distinguish between these two possible scenarios we examined the tree of domains and domain combinations generated by Wang and Caetano-Anollés [80] and determined the actual age of the two-domain proteins and the corresponding single domain domains. For example, the two domains of L2 have different *nd_P_* in the tree of domain structures (L2-N with the b.40.4 domain structure, *nd*_P_ = 0; L2-C with the b.34.5 structure, *nd*_P_ = 0.29). Using the published tree of domain combinations at FSF level, we find that the b.40.4|b.34.5 combination in L2 is younger (*nd*_P_ = 0.306) than domain b.40.4 of L2-N (*nd*_P_ = 0.037) but older than domain b.34.5 of L2-C (*nd*_P_ = 0.347) and its permutation b.34.5|b.40.4 (*nd*_P_ = 0.801). Consequently, the older domain was co-opted and the age of the L2 fusional-fissional combination is assigned the age of the younger domain in the tree of domain structures, i.e. the *nd*_P_ of L2-C. Similar rationale was used for other rearrangement scenarios. When this information was not available we assigned the age of the younger domain from the tree of domain structures (Figure 4) since the domain fusion in this case could not have occurred until the appearance of the newer protein.

Since protein interactions follow a linear correspondence with the age of rRNA helices until roughly the time of the second transition, after which there is rapid burst in the discovery of the new FSFs (Figure 4), we linked the age of rRNA helices (*nd*) with the age of r-proteins (*nd*_P_) that appeared late in evolution by plotting *nd* vs. *nd*_P_ and interpolating interactions (Figure 4).

### Construction of Evolutionary Heat Maps

To better visualize the relative age of the different elements of the ribosomal ensemble and to understand how the functions were associated with these structural elements, secondary structures of *Thermus thermophillus* rRNA corresponding to the crystal structure of the 70S ribosome (PDB entries 1GIX and GIY) and crystal structures of rRNA or the ribosomal ensemble of the *T. thermophillus* 70S ribosome (PDB entries 2WDK and 2WDL) were painted with colors corresponding to the age of rRNA helices (*nd*) and/or r-proteins (*nd*_P_) and visualized with standard molecular visualization software. An RGB color scale corresponding to the *nd* values 0–1 with an interval of 0.01 was produced in matplotlib [121] using scripts available at http://matplotlib.sourceforge.net/gallery.html and used to color the secondary structure models. While the crystal structures were similarly colored, the FSFs of r-proteins represent only a small subset of the structures in the tree of FSF domains, and r-protein *nd*_P_ values (range *nd*_P_ = 0.018–0.534) were normalized using a perl script to a 0–1 time scale for the color scale. Finally, 3D evolutionary heat maps were visualized using the UCSF Chimera package from the Resource for Biocomputing, Visualization, and Informatics at the University of California, San Francisco [122], [123], [124].

## Supporting Information

Figure S1 **Evolution of rRNA structure in individual rRNA subunits.** Universal trees of SSU rRNA helices (39,136 steps; CI = 0.835, RI = 0.971; HI = 0.165; g_1_ = −192.8) and LSU rRNA helices (138,582 steps; CI = 0.265, RI = 0.751; HI = 0.735; g_1_ = −24.5) were reconstructed from structural data in 19,184 and 593 ErDB sequences, respectively. Single most parsimonious trees were retained after a heuristic search with TBR branch swapping and simple addition sequence in both instances. The topology of trees is congruent with corresponding subtrees reconstructed from data used to build the tree of SSU and LSU rRNA helices of Figure 2. Topological congruence measured using several tree comparison metrics and randomization tools implemented in component reject a topological match by chance (*p*<0.01). For example, trees of SSU rRNA helices generated from the 19,184 ErDB sequences and the 93 sequence sets were mostly congruent (partition distance, PD = 60; symmetric difference, SD = 0.118 and SD = 0.179 for triplet and quartet analysis, respectively). The symmetric difference of Robinson and Foulds also supported significant topological congruence between trees (60 and 185 for SSU and LSU trees, respectively). Nodes with boostrap support (BS) values >50% are labeled.
(PDF)

Figure S2 **Comparison of the phylogenetic model (PM) and the A-minor interaction model (AM) of ribosome evolution.** A chronological representation of the evolution of the LSU rRNA shows that our PM based on a phylogeny of both LSU and SSU rRNA structure generally agrees with the AM based solely on the analysis of A-minor interactions in LSU rRNA ([31] in Text S2). The relative age of the LSU rRNA segments (*nd*) was divided into five time points corresponding to the number of stages in AM. Accretion is indicated by the number of LSU segments added at each stage of evolution. Except for the components involved in ribosomal processivity, PM matches AM in general. The PTC is highlighted in a lighter shade of its corresponding *nd*. The helix marked with an asterisk in PM that appears late in AM does not have an *nd* value since it is bacteria-specific and was not included in the phylogeny. The SSU rRNA is shaded in grey.
(PDF)

Figure S3 **Correspondence between the age of r-proteins and the age of first interacting rRNA helix.** The FSFs of r-proteins represents a small subset of FSFs that are known with *nd*_P_ values within the range 0.018–0.534. A method of interpolation was used to determine the age of r-proteins (*nd_P_*) with reference to the age of the interacting rRNA helix (*nd*). Figure shows that the protein interactions follow a linear correspondence with rRNA helices. Starting from the oldest protein and first interacting helix, the correspondence is maintained until the point of the second transition after which there is a rapid burst in the discovery of new FSFs. Hence the pattern of *nd_P_* and *nd* correspondence is interrupted. To determine the correspondence between the youngest r-proteins and the youngest rRNA helices, we interpolated their *nd_P_* values on the slope. *nd_P_* and *nd* values are given for all universal r-proteins.
(PDF)

Figure S4 **Evolutionary heat map showing the relative age of SSU and LSU r-proteins in the entire ribosomal ensemble.** The right panel is rotated by 180 degrees with respect to the left panel. The rRNA helices are colored according to their respective *nd* as in fig. 1 and r-proteins are colored according to their respective *nd*_P_ as in Figure 5. The r-protein *nd*_P_ were rescaled to a 0–1 scale as explained in Figure S2. This shows that older r-proteins are associated with older rRNA helices. The oldest r-proteins S12, S17, L3 and L2 are associated with the oldest rRNA helices involved in processivity and PTC. Most of the newer proteins are at the periphery of the functional assembly.
(PDF)

Figure S5 **Overview of the reconstruction of hypothetical ancestral sequences and structures from rRNA.** The flow chart describes the methods and data used to reconstruct ancestral rRNA molecules for remote homology analyses.
(PDF)

Figure S6 **Structural similarity of hypothetical rRNA helices and** ***in vitro*** **evolved ribozyme doppelgangers.** Results from the complete alignment experiment are presented. Plots with alignment scores (top panels) and Z-scores (bottom panels) are shown for all substructures in the three ribozymes and the control natural RNA molecule that were analyzed. Z-scores were derived from the alignment of 1,000 randomized sequences. Alignment scores of structures with Z-scores over 3 (horizontal dashed line) are significant at 0.01% confidence levels and significant matches in top panels are colored in red.
(PDF)

Figure S7 **Possible scenarios and likelihood of origins of ribosomal functions.** The evolutionary path leading to the emergence of translation is likely to be complex, requiring the discovery of multiple evolutionary novelties. Important among these novelties are the capacity to copy molecules and genetically encode products (pro) and the ability to biosynthesize complex polymers (bio). Such innovations are here envisioned as a natural outcome of primordial chemistries and under this scenario, the *de novo* appearance of complex functions is highly unlikely. Similarly, it is highly unlikely that a multi-component molecular complex harboring several functional processes needed for modern translation could emerge in a single or only a few events of evolutionary novelty. Instead, it is more likely that the evolution of ribosomal functions developed progressively by slow accretion of molecular structures that preexisted in other molecular contexts. Translation involves multiple mechanistic and functional steps and multiple players other than the ribosome, which could have been gradually recruited from simpler pre-existent molecular components (pro’, bio’) to perform a related but mechanistically more complex functional task. Results presented in this study are consistent with this gradual evolutionary scenario. While recruitment would have been combined with processes of gradual molecular evolution, crucial revolutionary transitions would have favored the functional emergence process by replacing the nonribosomally synthesized polypeptides with much improved analogs. Replacement of ancient nonribosomal protein synthetases that do not use a template to synthesize proteins or their precursors and the recruitment of ancient replication components for modern processivity and templating functions are most likely and are compatible with the diagrams of the figure.
(PDF)

Figure S8 **Testing assumptions for character state change.** All possible character changes were traced on the tree of rRNA helical elements of Figure 2, revealing how character state change distributes in trees of substructures of SSU and LSU rRNA.
(PDF)

Table S1 **rRNA helices and their associated functions.**
(PDF)

Table S2 **Order of establishment of intersubunit bridges and the rRNA helices and r- proteins involved in bridge interactions.**
(PDF)

Table S3 **FSFs of r-protein domains and their relative age.**
(PDF)

Table S4 **Age of rRNA helices (** ***nd*** **) interacting with r-proteins and the number of interacting rRNA residues.**
(PDF)

Table S5 **List of species from which sequences of both SSU and LSU rRNA were used for the reconstruction of the trees of rRNA helices shown in** **Figure 2** **.**
(PDF)

Text S1 **Phylogenetic analysis of molecular structure.**
(DOC)

Text S2 **Evolution of the functional rRNA core.**
(PDF)

Text S3 **Role of tRNA in ribosomal evolution.**
(PDF)

Text S4 **Origin and evolution of r-proteins.**
(PDF)

Text S5 **Assessing structural similarity to detect functional shifts.**
(PDF)
