# Supplementary material for: Ribosomal History Reveals Origins of Modern Protein Synthesis
Source: PLoS One. 2012 Mar 12;7(3):e32776. doi: 10.1371/journal.pone.0032776 (PMC3299690; doi:10.1371/journal.pone.0032776)
Supplement: Figure S1 — Evolution of rRNA structure in individual rRNA subunits. Universal trees of SSU rRNA helices (39,136 steps; CI = 0.835, RI = 0.971; HI = 0.165; g1 = −192.8) and LSU rRNA helices (138,582 steps; CI = 0.265, RI = 0.751; HI = 0.735; g1 = −24.5) were reconstructed from structural data in 19,184 and 593 ErDB sequences, respectively. Single most parsimonious trees were retained after a heuristic search with TBR branch swapping and simple addition sequence in both instances. The topology of trees is congruent with corresponding subtrees reconstructed from data used to build the tree of SSU and LSU rRNA helices of Figure 2. Topological congruence measured using several tree comparison metrics and randomization tools implemented in component reject a topological match by chance (p<0.01). For example, trees of SSU rRNA helices generated from the 19,184 ErDB sequences and the 93 sequence sets were mostly congruent (partition distance, PD = 60; symmetric difference, SD = 0.118 and SD = 0.179 for triplet and quartet analysis, respectively). The symmetric difference of Robinson and Foulds also supported significant topological congruence between trees (60 and 185 for SSU and LSU trees, respectively). Nodes with boostrap support (BS) values >50% are labeled. (PDF) [file pone.0032776.s001.pdf]

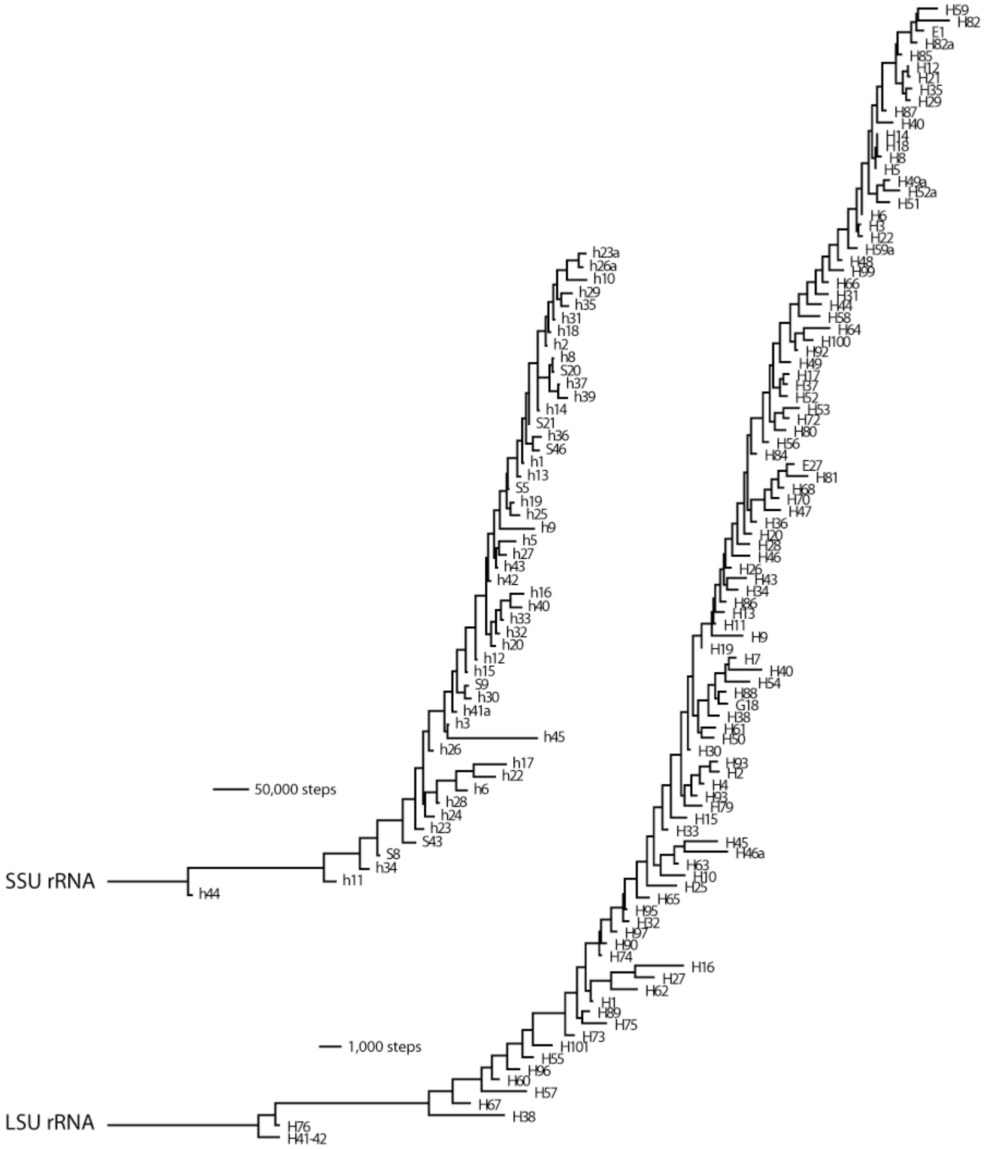

Figure S1. Evolution of rRNA structure in individual rRNA subunits. Universal trees of SSU rRNA helices (39,136 steps; CI = 0.835, RI = 0.971; HI = 0.165; g_1 = -192.8) and LSU rRNA helices (138,582 steps; CI = 0.265, RI = 0.751; HI = 0.735; g_1 = -24.5) were reconstructed from structural data in 19,184 and 593 ErDB sequences, respectively. Single most parsimonious trees were retained after a heuristic search with TBR branch swapping and simple addition sequence in both instances. The topology of trees is congruent with corresponding subtrees reconstructed from data used to build the tree of SSU and LSU rRNA helices of Figure 2. Topological congruence measured using several tree comparison metrics and randomization tools implemented in COMPONENT reject a topological match by chance ($p < 0.01$). For example, trees of SSU rRNA helices generated from the 19,184 ErDB sequences and the 93 sequence sets were mostly congruent (partition distance, PD = 60; symmetric difference, SD = 0.118 and SD = 0.179 for triplet and quartet analysis, respectively). The symmetric difference of Robinson and Foulds also supported significant topological congruence between trees (60 and 185 for SSU and LSU trees, respectively). Nodes with boostrap support (BS) values > 50% are labeled.
