# Supplementary material for: Ribosomal History Reveals Origins of Modern Protein Synthesis
Source: PLoS One. 2012 Mar 12;7(3):e32776. doi: 10.1371/journal.pone.0032776 (PMC3299690; doi:10.1371/journal.pone.0032776)
Supplement: Figure S2 — Comparison of the phylogenetic model (PM) and the A-minor interaction model (AM) of ribosome evolution. A chronological representation of the evolution of the LSU rRNA shows that our PM based on a phylogeny of both LSU and SSU rRNA structure generally agrees with the AM based solely on the analysis of A-minor interactions in LSU rRNA ([31] in Text S2). The relative age of the LSU rRNA segments (nd) was divided into five time points corresponding to the number of stages in AM. Accretion is indicated by the number of LSU segments added at each stage of evolution. Except for the components involved in ribosomal processivity, PM matches AM in general. The PTC is highlighted in a lighter shade of its corresponding nd. The helix marked with an asterisk in PM that appears late in AM does not have an nd value since it is bacteria-specific and was not included in the phylogeny. The SSU rRNA is shaded in grey. (PDF) [file pone.0032776.s002.pdf]

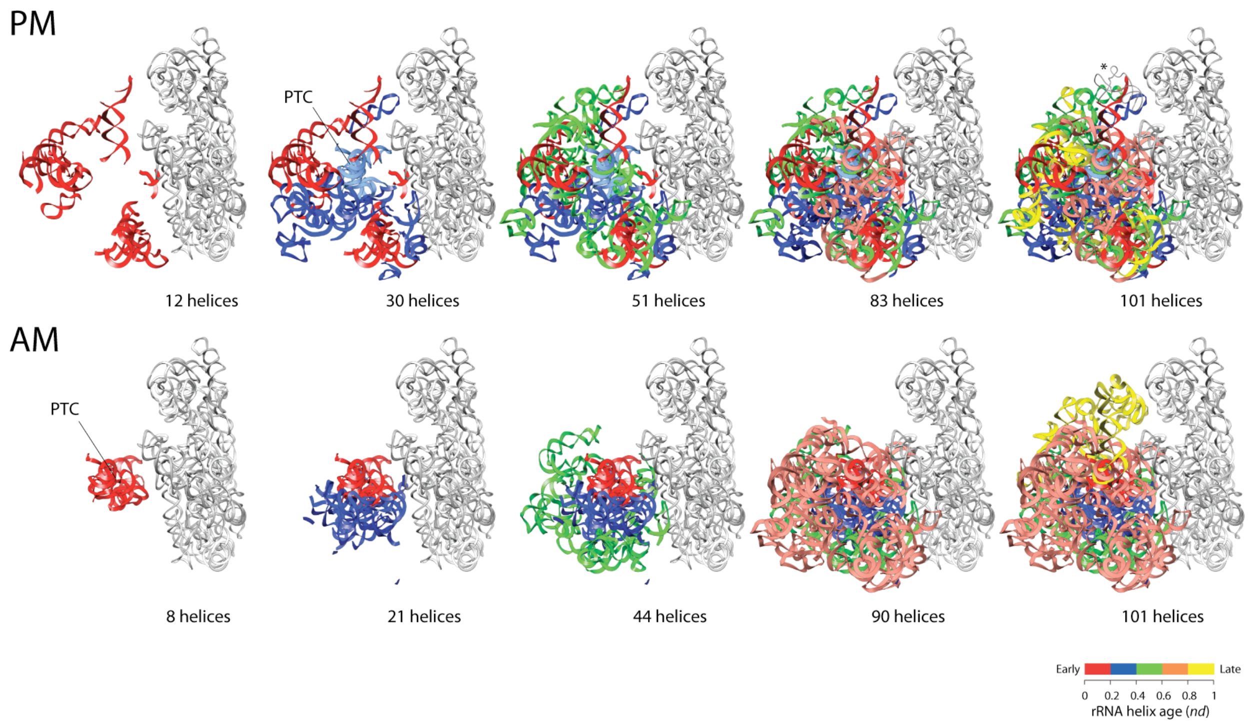

Figure S2. Comparison of the phylogenetic model (PM) and the A-minor interaction model (AM) of ribosome evolution. A chronological representation of the evolution of the LSU rRNA shows that our PM based on a phylogeny of both LSU and SSU rRNA structure generally agrees with the AM based solely on the analysis of A-minor interactions in LSU rRNA ([31] in Text S2). The relative age of the LSU rRNA segments (*nd*) was divided into five time points corresponding to the number of stages in AM. Accretion is indicated by the number of LSU segments added at each stage of evolution. Except for the components involved in ribosomal processivity, PM matches AM in general. The PTC is highlighted in a lighter shade of its corresponding *nd*. The helix marked with an asterisk in PM that appears late in AM does not have an *nd* value since it is bacteria-specific and was not included in the phylogeny. The SSU rRNA is shaded in grey.
