# Supplementary material for: Ribosomal History Reveals Origins of Modern Protein Synthesis
Source: PLoS One. 2012 Mar 12;7(3):e32776. doi: 10.1371/journal.pone.0032776 (PMC3299690; doi:10.1371/journal.pone.0032776)
Supplement: Figure S3 — Correspondence between the age of r-proteins and the age of first interacting rRNA helix. The FSFs of r-proteins represents a small subset of FSFs that are known with nd P values within the range 0.018–0.534. A method of interpolation was used to determine the age of r-proteins (ndP) with reference to the age of the interacting rRNA helix (nd). Figure shows that the protein interactions follow a linear correspondence with rRNA helices. Starting from the oldest protein and first interacting helix, the correspondence is maintained until the point of the second transition after which there is a rapid burst in the discovery of new FSFs. Hence the pattern of ndP and nd correspondence is interrupted. To determine the correspondence between the youngest r-proteins and the youngest rRNA helices, we interpolated their ndP values on the slope. ndP and nd values are given for all universal r-proteins. (PDF) [file pone.0032776.s003.pdf]

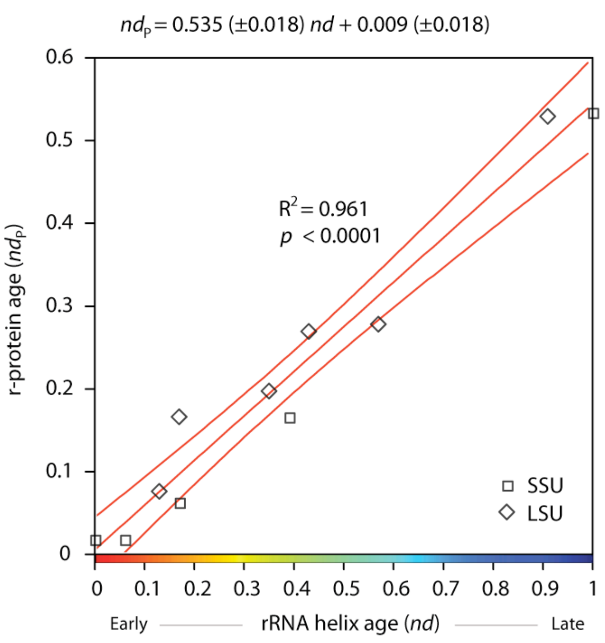

	r-proteins	nd_P	nd
32	S15	0.534	1.00
31	L23	0.259	0.99
30	L15	0.525	0.98
29	L30	0.516	0.97
28	L1	0.516	0.97
27	L4	0.507	0.95
26	L22	0.502	0.94
25	L11	0.502	0.94
24	S10	0.493	0.92
23	L13	0.493	0.92
22	L14	0.489	0.91
21	S19	0.480	0.90
20	S2	0.480	0.90
19	L5	0.480	0.90
18	S8	0.475	0.89
17	S7	0.475	0.89
16	S3	0.457	0.85
15	L29	0.457	0.85
14	L7/L12	0.417	0.77
13	S5	0.291	0.53
12	S11	0.278	0.51
11	L18	0.278	0.51
10	S14	0.269	0.49
9	L6	0.269	0.49
8	S13	0.260	0.47
7	S4	0.197	0.35
6	L24	0.166	0.29
5	L2	0.166	0.29
4	L3	0.076	0.11
3	S9	0.063	0.09
2	S17	0.018	0.00
1	S12	0.018	0.00

Figure S3. Correspondence between the age of r-proteins and the age of first interacting rRNA helix. The FSFs of r-proteins represents a small subset of FSFs that are known with nd_P values within the range 0.018-0.534. A method of interpolation was used to determine the age of r-proteins (nd_P) with reference to the age of the interacting rRNA helix (*nd*). Figure shows that the protein interactions follow a linear correspondence with rRNA helices. Starting from the oldest protein and first interacting helix, the correspondence is maintained until the point of the second transition after which there is a rapid burst in the discovery of new FSFs. Hence the pattern of nd_P and *nd* correspondence is interrupted. To determine the correspondence between the youngest r-proteins and the youngest rRNA helices, we interpolated their nd_P values on the slope. nd_P and *nd* values are given for all universal r-proteins.
