# Supplementary material for: Ribosomal History Reveals Origins of Modern Protein Synthesis
Source: PLoS One. 2012 Mar 12;7(3):e32776. doi: 10.1371/journal.pone.0032776 (PMC3299690; doi:10.1371/journal.pone.0032776)
Supplement: Figure S4 — Evolutionary heat map showing the relative age of SSU and LSU r-proteins in the entire ribosomal ensemble. The right panel is rotated by 180 degrees with respect to the left panel. The rRNA helices are colored according to their respective nd as in fig. 1 and r-proteins are colored according to their respective nd P as in Figure 5. The r-protein nd P were rescaled to a 0–1 scale as explained in Figure S2. This shows that older r-proteins are associated with older rRNA helices. The oldest r-proteins S12, S17, L3 and L2 are associated with the oldest rRNA helices involved in processivity and PTC. Most of the newer proteins are at the periphery of the functional assembly. (PDF) [file pone.0032776.s004.pdf]

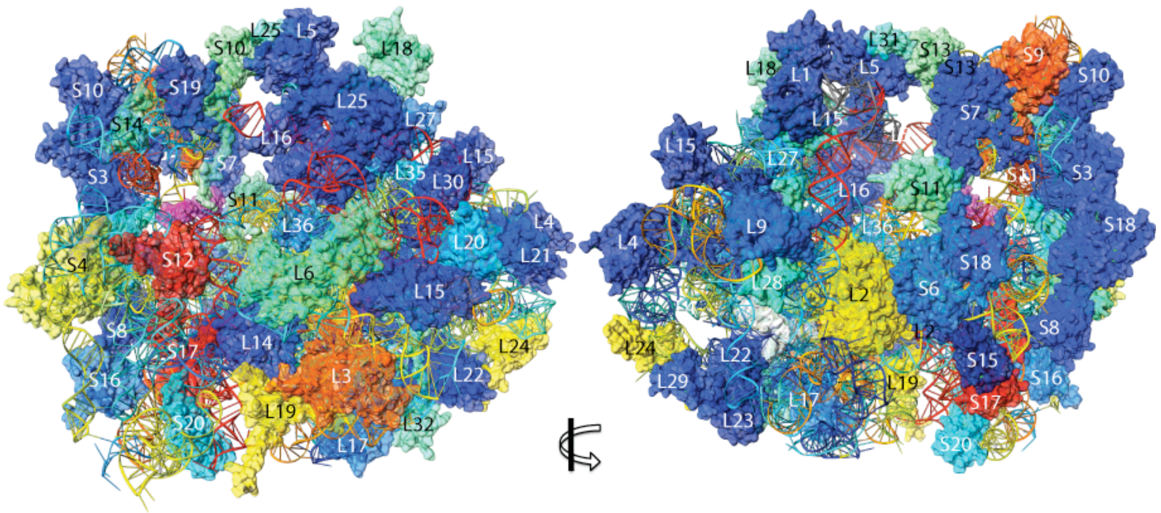

Figure S4. Evolutionary heat map showing the relative age of SSU and LSU r-proteins in the entire ribosomal ensemble. The right panel is rotated by 180 degrees with respect to the left panel. The rRNA helices are colored according to their respective *nd* as in fig. 1 and r-proteins are colored according to their respective nd_P as in Figure 5. The r-protein nd_P were rescaled to a 0-1 scale as explained in Figure S3. This shows that older r-proteins are associated with older rRNA helices. The oldest r-proteins S12, S17, L3 and L2 are associated with the oldest rRNA helices involved in processivity and PTC. Most of the newer proteins are at the periphery of the functional assembly.
