# Supplementary material for: Ribosomal History Reveals Origins of Modern Protein Synthesis
Source: PLoS One. 2012 Mar 12;7(3):e32776. doi: 10.1371/journal.pone.0032776 (PMC3299690; doi:10.1371/journal.pone.0032776)
Supplement: Figure S5 — Overview of the reconstruction of hypothetical ancestral sequences and structures from rRNA. The flow chart describes the methods and data used to reconstruct ancestral rRNA molecules for remote homology analyses. (PDF) [file pone.0032776.s005.pdf]

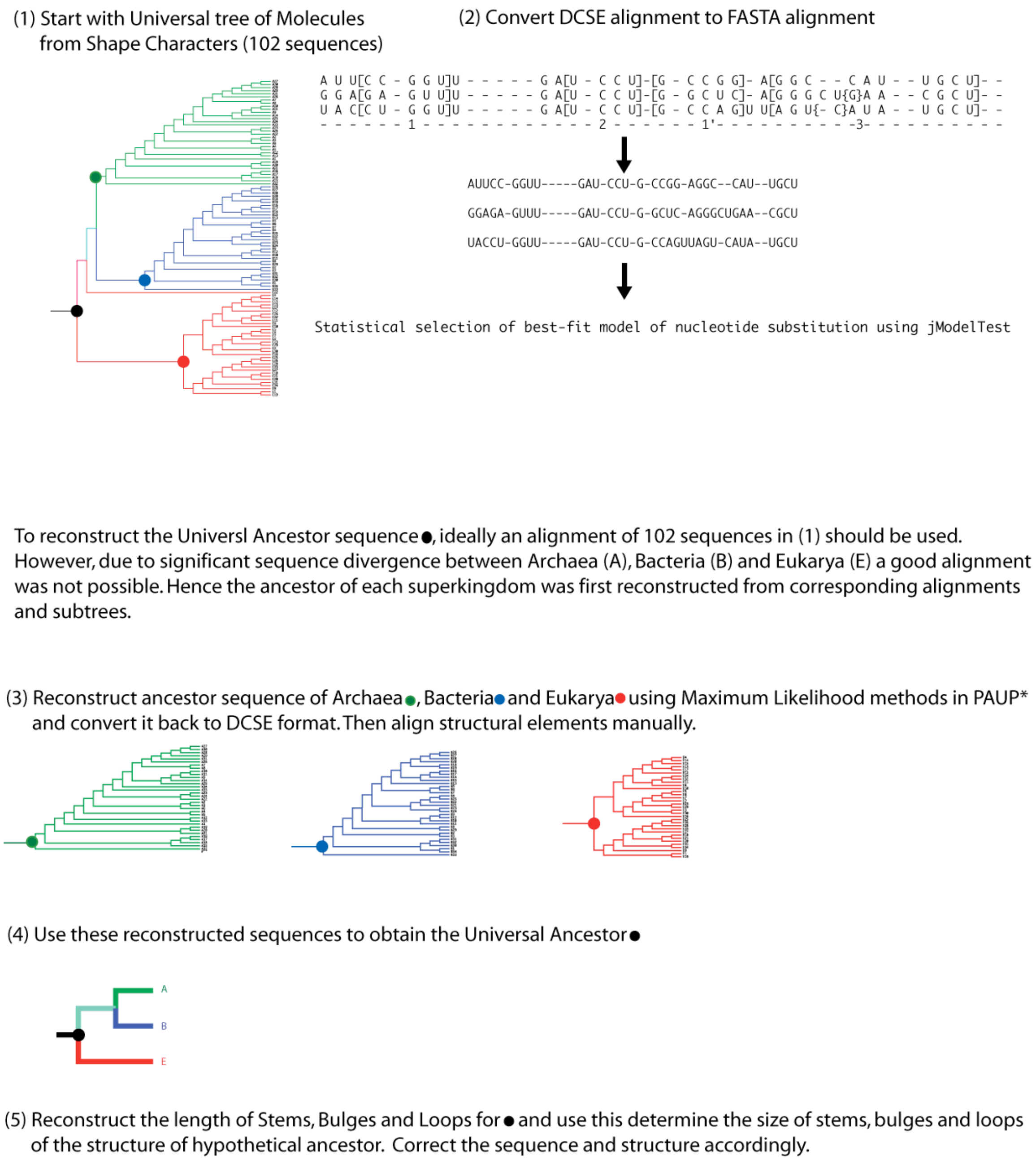

Figure S5. Overview of the reconstruction of hypothetical ancestral sequences and structures from rRNA. The flow chart describes the methods and data used to reconstruct ancestral rRNA molecules for remote homology analyses.
