# Supplementary material for: Ribosomal History Reveals Origins of Modern Protein Synthesis
Source: PLoS One. 2012 Mar 12;7(3):e32776. doi: 10.1371/journal.pone.0032776 (PMC3299690; doi:10.1371/journal.pone.0032776)
Supplement: Figure S6 — Structural similarity of hypothetical rRNA helices and in vitro evolved ribozyme doppelgangers. Results from the complete alignment experiment are presented. Plots with alignment scores (top panels) and Z-scores (bottom panels) are shown for all substructures in the three ribozymes and the control natural RNA molecule that were analyzed. Z-scores were derived from the alignment of 1,000 randomized sequences. Alignment scores of structures with Z-scores over 3 (horizontal dashed line) are significant at 0.01% confidence levels and significant matches in top panels are colored in red. (PDF) [file pone.0032776.s006.pdf]

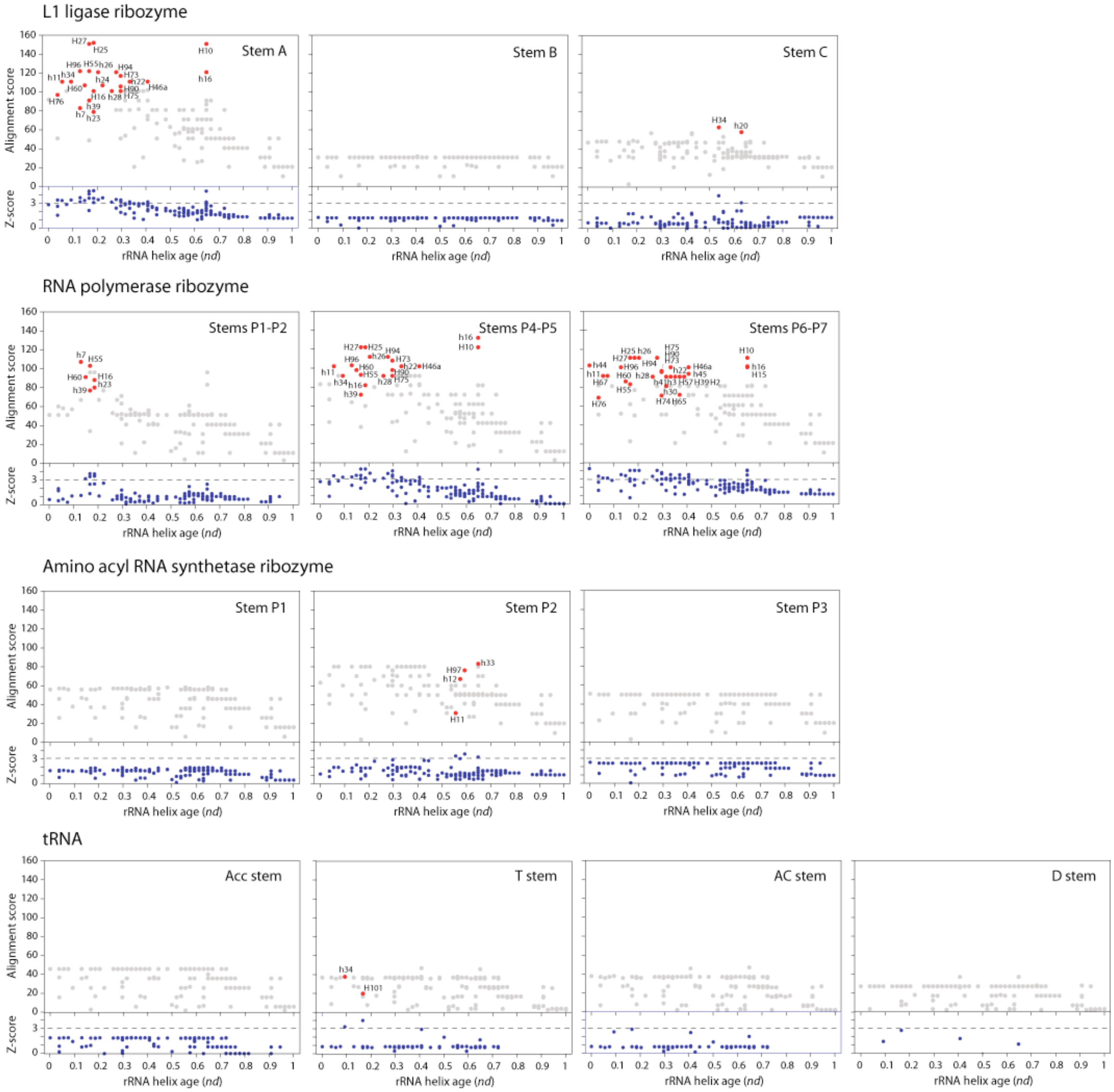

Figure S6. Structural similarity of hypothetical rRNA helices and *in vitro* evolved ribozyme doppelgangers. Results from the complete alignment experiment are presented. Plots with alignment scores (top panels) and Z-scores (bottom panels) are shown for all substructures in the three ribozymes and the control natural RNA molecule that were analyzed. Z-scores were derived from the alignment of 1,000 randomized sequences. Alignment scores of structures with Z-scores over 3 (horizontal dashed line) are significant at 0.01% confidence levels and significant matches in top panels are colored in red.
