# Supplementary material for: Ribosomal History Reveals Origins of Modern Protein Synthesis
Source: PLoS One. 2012 Mar 12;7(3):e32776. doi: 10.1371/journal.pone.0032776 (PMC3299690; doi:10.1371/journal.pone.0032776)
Supplement: Figure S7 — Possible scenarios and likelihood of origins of ribosomal functions. The evolutionary path leading to the emergence of translation is likely to be complex, requiring the discovery of multiple evolutionary novelties. Important among these novelties are the capacity to copy molecules and genetically encode products (pro) and the ability to biosynthesize complex polymers (bio). Such innovations are here envisioned as a natural outcome of primordial chemistries and under this scenario, the de novo appearance of complex functions is highly unlikely. Similarly, it is highly unlikely that a multi-component molecular complex harboring several functional processes needed for modern translation could emerge in a single or only a few events of evolutionary novelty. Instead, it is more likely that the evolution of ribosomal functions developed progressively by slow accretion of molecular structures that preexisted in other molecular contexts. Translation involves multiple mechanistic and functional steps and multiple players other than the ribosome, which could have been gradually recruited from simpler pre-existent molecular components (pro’, bio’) to perform a related but mechanistically more complex functional task. Results presented in this study are consistent with this gradual evolutionary scenario. While recruitment would have been combined with processes of gradual molecular evolution, crucial revolutionary transitions would have favored the functional emergence process by replacing the nonribosomally synthesized polypeptides with much improved analogs. Replacement of ancient nonribosomal protein synthetases that do not use a template to synthesize proteins or their precursors and the recruitment of ancient replication components for modern processivity and templating functions are most likely and are compatible with the diagrams of the figure. (PDF) [file pone.0032776.s007.pdf]

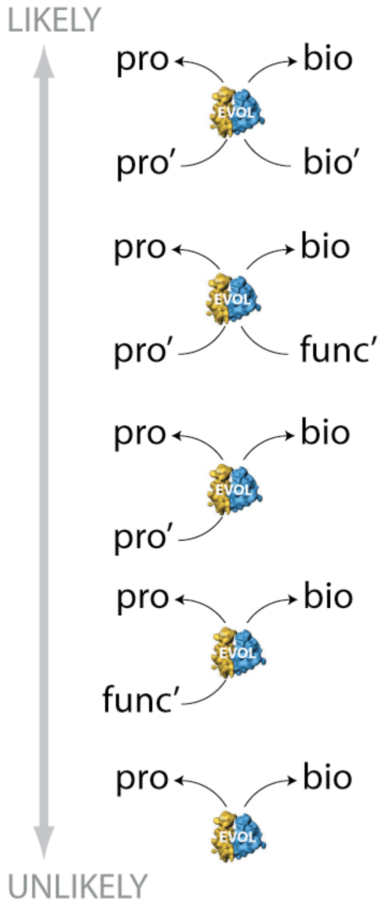

Figure S7. Possible scenarios and likelihood of origins of ribosomal functions. The evolutionary path leading to the emergence of translation is likely to be complex, requiring the discovery of multiple evolutionary novelties. Important among these novelties are the capacity to copy molecules and genetically encode products (pro) and the ability to biosynthesize complex polymers (bio). Such innovations are here envisioned as a natural outcome of primordial prebiotic chemistries and under this scenario, the *de novo* appearance of complex functions is highly unlikely. Similarly, it is highly unlikely that a multi-component molecular complex harboring several functional processes needed for modern translation could emerge in a single or only a few events of evolutionary novelty. Instead, it is more likely that the evolution of ribosomal functions developed progressively by slow accretion of molecular structures that preexisted in other molecular contexts. Translation involves multiple mechanistic and functional steps and multiple players other than the ribosome, which could have been gradually recruited from simpler pre-existent molecular components (pro', bio') to perform a related but mechanistically more complex functional task. Results presented in this study are consistent with this gradual evolutionary scenario. While recruitment would have been combined with processes of gradual molecular evolution, crucial revolutionary transitions would have favored the functional emergence process by replacing the nonribosomally synthesized polypeptides with much improved analogs. Replacement of ancient nonribosomal protein synthetases that do not use a template to synthesize proteins or their precursors and the recruitment of ancient replication components for modern processivity and templating functions are most likely and are compatible with the diagrams of the figure.
