# Supplementary material for: Ribosomal History Reveals Origins of Modern Protein Synthesis
Source: PLoS One. 2012 Mar 12;7(3):e32776. doi: 10.1371/journal.pone.0032776 (PMC3299690; doi:10.1371/journal.pone.0032776)
Supplement: Figure S8 — Testing assumptions for character state change. All possible character changes were traced on the tree of rRNA helical elements of Figure 2, revealing how character state change distributes in trees of substructures of SSU and LSU rRNA. (PDF) [file pone.0032776.s008.pdf]

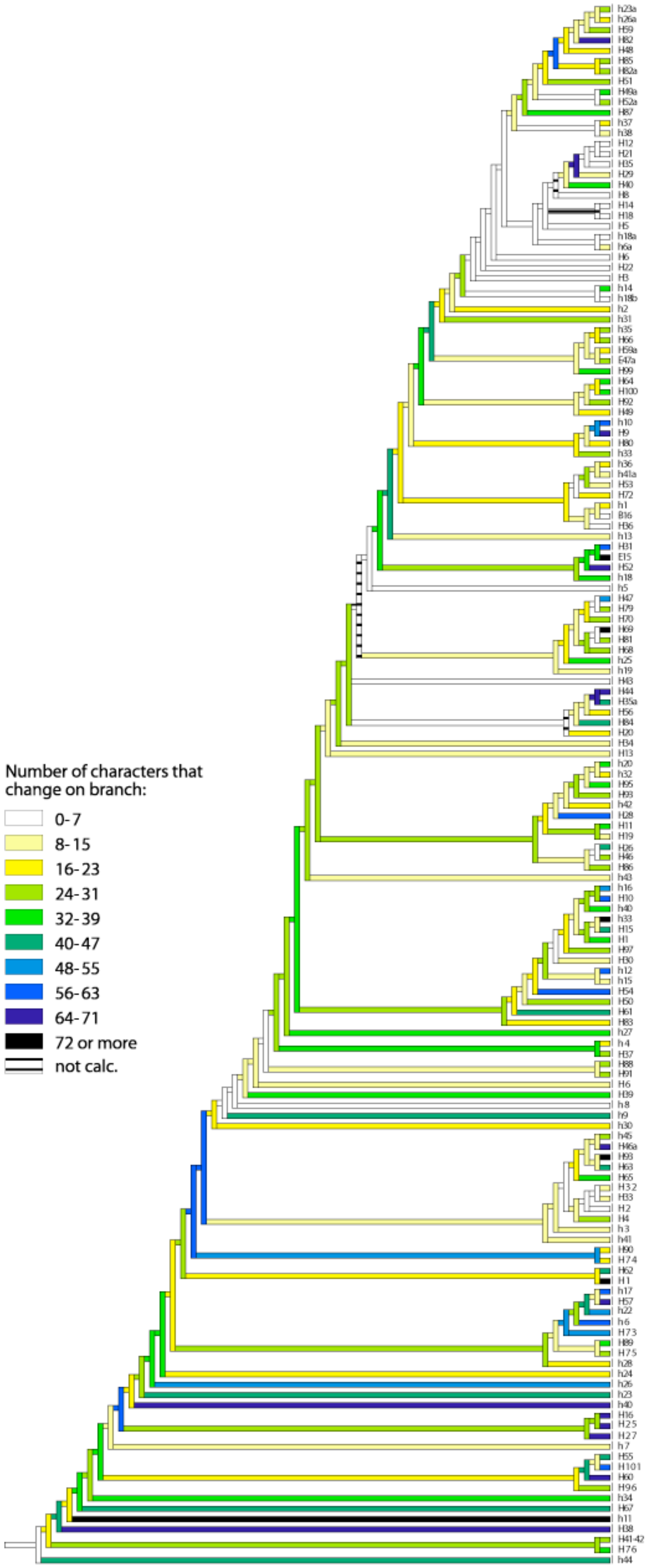

Figure S8. Testing assumptions for character state change. All possible character changes were traced on the tree of rRNA helical elements of Figure 2, revealing how character state change distributes in trees of substructures of SSU and LSU rRNA.
