# Supplementary material for: Ribosomal History Reveals Origins of Modern Protein Synthesis
Source: PLoS One. 2012 Mar 12;7(3):e32776. doi: 10.1371/journal.pone.0032776 (PMC3299690; doi:10.1371/journal.pone.0032776)
Supplement: Table S1 — rRNA helices and their associated functions. (PDF) [file pone.0032776.s009.pdf]

Table S1. rRNA helices and their associated functions

Helix number		Subunit		*nd*		Functional centers				tRNA interactions		Bridge interactions	
Brima-combe	ErDB		Age	mRNA deco-ding	mRNA helic-case	tRNA trans-loca-tion	GTPase center	PTC	AC stem	T stem	D stem	Acc stem	
h44	S49	SSU	0.000	+	+				+				B5, B6, B3, B2a
H38	D14	LSU	0.037			+				+			
H41	D17	LSU	0.037										
H42	D17	LSU	0.037				+						
H76	G4	LSU	0.037			+							
h11	S12	SSU	0.056										
H67	E25	LSU	0.074										B2b, B2c
h34	S38	SSU	0.093		+	+			+				
h7	S8	SSU	0.130										
H96	H3	LSU	0.130										
H60	E18	LSU	0.148										
H101	J3	SSU	0.167										
H27	D2	LSU	0.167										B5
h39	S43	SSU	0.167										
H55	E12	LSU	0.167										
H16	B15	LSU	0.185										
h23	S25	SSU	0.185						+				B7b, B7a
H25	C1	LSU	0.185										
h26	S29	SSU	0.204										
h24	S27	SSU	0.222						+	+	+		B7b
h28	S32	SSU	0.259		+				+				
H62	E20	LSU	0.278										B5, B6
H94	H1	LSU	0.278					+					
H73	G1	LSU	0.296					+					
H74	G2	LSU	0.296					+				+	
H75	G3	LSU	0.296									+	
H89	G16	LSU	0.296					+		+		+	
H90	G17	LSU	0.296					+					
h30	S34	SSU	0.315						+				
h41	S45	SSU	0.315										
h6	S6	SSU	0.315										
h22	S24	SSU	0.333										B7b
h3	S3	SSU	0.333										
h9	S10	SSU	0.333										
h17	S18	SSU	0.352										
H57	E14	LSU	0.352										
h8	S9	SSU	0.352										
H39	D15	LSU	0.370							+			
H4	B3	LSU	0.370										

H65	E23	LSU	0.370										
H2	B1	LSU	0.389										
H7	B6	LSU	0.389										
H32	D7	LSU	0.407										
H33	D8	LSU	0.407										
H45	D20	LSU	0.407										
h45	S50	SSU	0.407										B2b
H46a	D22	LSU	0.407										
H63	E21	LSU	0.407										
H88	G15	LSU	0.426										
H91	G18	LSU	0.426										
h27	S31	SSU	0.444										B2c
H37	D13	LSU	0.444										
h4	S4	SSU	0.444										
h43	S48	SSU	0.481										
H83	G10	LSU	0.481										
H61	E19	LSU	0.500										
H13	B12	LSU	0.519										
H50	E6	LSU	0.519										
H34	D9	LSU	0.537										B4
H54	E11	LSU	0.537										
H86	G13	LSU	0.537										
H11	B10	LSU	0.556										
H19	B18	LSU	0.556										
H20	B19	LSU	0.556										
H26	D1	LSU	0.556										
H28	D3	LSU	0.556										
H46	D21	LSU	0.556										
h12	S13	SSU	0.574										
h15	S16	SSU	0.574										
H31	D5	LSU	0.574										
h42	S47	SSU	0.574										
H84	G11	LSU	0.574										
h19	S22	SSU	0.593										
h5	S5	SSU	0.593										
H56	E13	LSU	0.593										
H93	G20	LSU	0.593					+				+	
H97	H4	LSU	0.593										
h25	S28	SSU	0.611										
H35a	D11	LSU	0.611										
H95	H2	LSU	0.611										
H1	A1	LSU	0.630										
h13	S14	SSU	0.630										
h18	S19	SSU	0.630						+		+		
h20	S23	SSU	0.630										B4
h32	S36	SSU	0.630										
h40	S44	SSU	0.630										
H43	D18	LSU	0.630			+	+						
H44	D19	LSU	0.630			+	+						
H10	B9	LSU	0.648									+	
H15	B14	LSU	0.648										
h16	S17	SSU	0.648										

h33	S37	SSU	0.648										
H52	E8	LSU	0.648										
H68	E26	LSU	0.648			+						+	B7a
H70	E28	LSU	0.648										
H71	E29	LSU	0.648									+	B5,<br>B2b,<br>B3
H30	D6	LSU	0.667										
H47	E2	LSU	0.667										
H58	E15	LSU	0.667										
H69	E27	LSU	0.667			+					+		B2b,<br>B2a
H79	G5	LSU	0.667							+	+		
H81	G7	LSU	0.667										
h29	S33	SSU	0.685						+	+			
H36	D12	LSU	0.685										
H72	F1	LSU	0.685										
h1	S1	SSU	0.704										
H17	B16	LSU	0.704										
H49	E4	LSU	0.704										
H53	E10	LSU	0.704										
H80	G6	LSU	0.704										
h10	S11	SSU	0.722										
h31	S35	SSU	0.722						+				
h36	S40	SSU	0.722										
h41a	S46	SSU	0.722										
H9	B8	LSU	0.722										
H92	G19	LSU	0.722										
H99	J1	LSU	0.722										
H100	J2	LSU	0.741										
h2	S2	SSU	0.741										
H64	E22	LSU	0.741										B5
h35	S39	SSU	0.759										
H47a	E3	LSU	0.759										
H59a	E17	LSU	0.759										
H66	E24	LSU	0.759										B2c
h14	S15	SSU	0.778										B8
h18b	S21	SSU	0.778										
H3	B2	LSU	0.778										
H22	B21	LSU	0.796										
H6	B5	LSU	0.815										
h18a	S20	SSU	0.870										
h37	S41	SSU	0.870										
h38	S42	SSU	0.870										
H5	B4	LSU	0.870										
h6a	S7	SSU	0.870										
H87	G14	LSU	0.870										
H14	B13	LSU	0.889										
H18	B17	LSU	0.889										
H8	B7	LSU	0.889										
H40	D16	LSU	0.907										
H49a	E5	LSU	0.907										
H51	E7	LSU	0.907										

H52a	E9	LSU	0.907										
H29	D4	LSU	0.926										
H35	D10	LSU	0.944										
H48	E1	LSU	0.944										
H82a	G9	LSU	0.944										
H85	G12	LSU	0.944										
H12	B11	LSU	0.963										
H21	B20	LSU	0.963										
H82	G8	LSU	0.963										
H59	E16	LSU	0.981										
h23a	S26	SSU	1.000										
h26a	S30	SSU	1.000										
