# Supplementary material for: Ribosomal History Reveals Origins of Modern Protein Synthesis
Source: PLoS One. 2012 Mar 12;7(3):e32776. doi: 10.1371/journal.pone.0032776 (PMC3299690; doi:10.1371/journal.pone.0032776)
Supplement: Table S2 — Order of establishment of intersubunit bridges and the rRNA helices and r- proteins involved in bridge interactions. (PDF) [file pone.0032776.s010.pdf]

Table S2. Order of establishment of intersubunit bridges and the rRNA helices and r-proteins involved in bridge interactions. R is RNA, P is protein, h is SSU helix, H is LSU helix. Data from Gao et al. [74] and Kietrys et al. [75].

			SSU		LSU	
Age	Bridge	Type	rRNA	r-Protein	rRNA	r-Protein
1	B5	R-R<br>R-P	h44<br>h44		H27, H62, H64, H71	 L14
2	B2b	R-R	h24, h45		H67, H69, H71	
3	B2c	R-R	h24, h27		H66,H67	
4	B6	R-R<br>R-P	h44<br>h44		H62	 L19
5	B7b	R-P	h22, h23, h24			L2
6	B1a	P-R		S13, S19	H38	
7	B4	R-R<br>P-R	h20	 S15	H34<br>H34	
8	B7a	R-R	h23		H68	
9	B3	R-R	h44		H71	
10	B2a	R-R	h44		H69	
11	B1b	P-P		S13		L5
12	B8	R-P	h14			L14, L19
