# Supplementary material for: Ribosomal History Reveals Origins of Modern Protein Synthesis
Source: PLoS One. 2012 Mar 12;7(3):e32776. doi: 10.1371/journal.pone.0032776 (PMC3299690; doi:10.1371/journal.pone.0032776)
Supplement: Table S3 — FSFs of r-protein domains and their relative age. (PDF) [file pone.0032776.s011.pdf]

Table S3. FSFs of r-protein domains and their relative age. Highlighted cells are universal.

SCOP ID	SCOP *ccs*	r-protein	*nd*	*nd* norm	SCOP name
50249	b.40.4	**L2 -N**	0.018	0.00	Nucleic acid-binding proteins
50249	b.40.4	**S12**	0.018	0.00	Nucleic acid-binding proteins
50249	b.40.4	**S17**	0.018	0.00	Nucleic acid-binding proteins
54211	d.14.1	**S5 -C**	0.063	0.09	r-Protein S5 domain 2-like
54211	d.14.1	**S9**	0.063	0.09	r-Protein S5 domain 2-like
50447	b.43.3	**L3**	0.076	0.11	Translation proteins
50104	b.34.5	**L2 -C**	0.166	0.29	Translation proteins SH3-like domain
50104	b.34.5	**L19**	0.166	0.29	Translation proteins SH3-like domain
50104	b.34.5	**L24**	0.166	0.29	Translation proteins SH3-like domain
55174	d.66.1	**S4**	0.197	0.35	Alpha-L RNA-binding motif
54814	d.52.3	**S3 -N**	0.206	0.37	Prokaryotic type KH domain (KH-domain type II)
46946	a.156.1	**S13**	0.260	0.47	S13-like H2TH domain
56053	d.141.1	**L6**	0.269	0.49	r-Protein L6
57716	g.39.1	**S14**	0.269	0.49	Glucocorticoid receptor-like (DNA-binding domain)
53137	c.55.4	**L18**	0.278	0.50	Translational machinery components
53137	c.55.4	**S11**	0.278	0.50	Translational machinery components
57829	g.41.8	**L32p**	0.283	0.51	Zn-binding r-proteins
57829	g.41.8	**L33p**	0.283	0.51	Zn-binding r-proteins
143800	d.325.1	**L28**	0.291	0.53	L28p-like
143800	d.325.1	**L31p**	0.291	0.53	L28p-like
54768	d.50.1	**S5 -N**	0.291	0.53	dsRNA-binding domain-like
55315	d.79.3	**L7ae**	0.296	0.54	L30e-like
55653	d.99.1	**L9 -C**	0.350	0.64	r-Protein L9 C-domain
46992	a.7.6	**S20**	0.354	0.65	r-Protein S20
74731	a.144.2	**L20**	0.381	0.70	r-Protein L20
143034	d.301.1	**L35p**	0.395	0.73	L35p-like
48300	a.108.1	**L7**	0.417	0.77	r-Protein L7/12, oligomerisation (N-terminal) domain
48300	a.108.1	**L12**	0.417	0.77	r-Protein L7/12, oligomerisation (N-terminal) domain
57840	g.42.1	**L36**	0.417	0.77	r-Protein L36
64263	d.188.1	**L17**	0.422	0.78	Prokaryotic r-protein L17
54995	d.58.14	**S6**	0.422	0.78	r-Protein S6
54565	d.27.1	**S16**	0.422	0.78	r-Protein S16
55658	d.100.1	**L9 -N**	0.430	0.80	L9 N-domain-like
141091	b.155.1	**L21p**	0.430	0.80	L21p-like
46911	a.4.8	**S18**	0.435	0.81	r-Protein S18
46561	a.2.2	**L29**	0.457	0.85	r-Protein L29 (L29p)
54821	d.53.1	**S3 -C**	0.457	0.85	r-Protein S3 C-terminal domain
47973	a.75.1	**S7**	0.475	0.89	r-Protein S7
56047	d.140.1	**S8**	0.475	0.89	r-Protein S8
55282	d.77.1	**L5**	0.480	0.90	RL5-like
52313	c.23.15	**S2**	0.480	0.90	r-Protein S2
54570	d.28.1	**S19**	0.480	0.90	r-Protein S19
50193	b.39.1	**L14**	0.489	0.91	r-Protein L14
52161	c.21.1	**L13**	0.493	0.92	r-Protein L13
54686	d.41.4	**L16p**	0.493	0.92	r-Protein L16p/L10e
54999	d.58.15	**S10**	0.493	0.92	r-Protein S10
46906	a.4.7	**L11 -C**	0.502	0.94	r-Protein L11, C-terminal domain
54747	d.47.1	**L11 -N**	0.502	0.94	r-Protein L11/L12e N-terminal domain
54843	d.55.1	**L22**	0.502	0.94	r-Protein L22

52166	c.22.1	**L4**	0.507	0.95	r-Protein L4
50715	b.53.1	**L25**	0.507	0.95	r-Protein L25-like
56808	e.24.1	**L1**	0.516	0.97	r-Protein L1
55129	d.59.1	**L30**	0.516	0.97	r-Protein L30p/L7e
52080	c.12.1	**L15**	0.525	0.98	r-Proteins L15p and L18e
54189	d.12.1	**L23**	0.529	0.99	r-Proteins S24e, L23 and L15e
47060	a.16.1	**S15**	0.534	1.00	S15/NS1 RNA-binding domain
160369	d.58.62	**L10**	N/A		r-Protein L10-like
64659	j.84.1	**L10**	N/A		r-Protein L10
144321	j.118.1	**L34p**	N/A		r-Protein L34p
58322	j.9.1	**S THX**	N/A		30S r-Protein THX
