# Supplementary material for: Ribosomal History Reveals Origins of Modern Protein Synthesis
Source: PLoS One. 2012 Mar 12;7(3):e32776. doi: 10.1371/journal.pone.0032776 (PMC3299690; doi:10.1371/journal.pone.0032776)
Supplement: Table S4 — Age of rRNA helices ( nd ) interacting with r-proteins and the number of interacting rRNA residues. (PDF) [file pone.0032776.s012.pdf]

Table S4. Age of rRNA helices (*nd*) interacting with universal r-proteins and the number of interacting rRNA residues.

FSF	r-Protein	nd_P	rRNA contact		*nd*	Number of interacting bases in helices	
			Brimacombe	ErRD			
b.40.4	**S12**	0.018	h44	S49	0.000	2	47
			h3	S3	0.333	8	
			h27	S31	0.444	8	
			h19	S22	0.593	6	
			h5	S5	0.593	3	
			h18	S19	0.630	20	
b.40.5	**S17**	0.018	h11	S12	0.056	19	39
			h7	S8	0.130	10	
			h27	S31	0.444	2	
			h20	S23	0.593	7	
			h23/ab2	S23/ab2	N/A	1	
d.14.2	**S9**	0.063	h39	S43	0.167	8	54
			h30	S34	0.315	4	
			h41	S45	0.315	8	
			h43	S48	0.481	16	
			h40	S44	0.630	3	
			h29	S33	0.685	4	
			h38	S42	0.870	11	
b.43.3	**L3**	0.076	H96	H3	0.130	12	92
			H101	J3	0.167	4	
			H94	H1	0.278	26	
			H73	G1	0.296	12	
			H90	G17	0.296	14	
			H61	E19	0.500	14	
			H100	J2	0.741	10	
b.34.4	**L2**	0.166	H74	G2	0.296	6	79
			H75	G3	0.296	8	
			H33	D8	0.407	11	
			H93	G20	0.593	8	
			H35a	D11	0.611	6	
			H67	E26	0.648	8	
			H79	G5	0.667	2	
			H66	E24	0.759	30	
b.34.5	**L24**	0.166	H7	B6	0.389	13	19
			H19	B19	0.556	6	
d.66.1	**S4**	0.197	h17	S18	0.352	9	50
			h5	S5	0.593	4	
			h18	S19	0.630	16	
			h16	S17	0.648	15	
			h23a	S26	1.000	3	
			h23/ab1	S23/ab1	N/A	3	
a.156.1	**S13**	0.260	h30	S34	0.315	15	32
			h41	S45	0.315	3	
			h42	S47	0.574	11	
			h31	S35	0.722	3	
d.141.1	**L6**	0.269	H91	G18	0.426	7	23

			H97	H4	0.593	8	
			H95	H2	0.611	8	
g.39.1	**S14**	0.269	h43	S48	0.481	4	29
			h42	S47	0.574	2	
			h32	S36	0.630	3	
			h31	S35	0.722	7	
			h36	S40	0.722	4	
			h38	S42	0.870	7	
			h37/b1	S37/b1		2	
c.55.4	**L18**	0.278	H38	D14	0.037	2	10
			H85	G11	0.574	2	
			H87	G14	0.870	6	
c.55.4	**S11**	0.278	h23	S25	0.185	12	30
			h23	S25	0.185	9	
			h24	S27	0.222	6	
			h45	S50	0.407	3	
d.14.1	**S5**	0.291	h28	S32	0.259	4	21
			h1	S1	0.704	5	
			h36	S40	0.722	5	
			h35	S39	0.759	4	
			h35	S39	0.759	3	
c.21.1	**L13**	0.417	H25	C1	0.185	6	42
			H94	H1	0.278	6	
			H42	D18	0.630	22	
			H72	F1	0.685	8	
a.2.2	**L29**	0.457	H7	B6	0.389	13	13
d.53.1	**S3**	0.457	h34	S38	0.093	24	41
			h16	S17	0.648	1	
			h36	S40	0.722	7	
			h35	S39	0.759	4	
			h38	S42	0.870	3	
			h37	S41	0.870	2	
a.75.1	**S7**	0.475	h28	S32	0.259	8	62
			h30	S34	0.315	10	
			h41	S45	0.315	16	
			h43	S48	0.481	18	
			h42	S47	0.574	7	
			h29	S33	0.685	3	
d.140.1	**S8**	0.475	h26	S29	0.204	4	47
			h25	S28	0.611	15	
			h23/ab1	S23/ab1		28	
d.77.1	**L5**	0.480	H84	G11	0.574	16	16
c.23.15	**S2**	0.480	h34	S38	0.093	1	19
			h26	S29	0.204	2	
			h25	S28	0.611	6	
			h36	S40	0.722	3	
			h35	S39	0.759	2	
			h37	S41	0.870	5	
d.28.1	**S19**	0.480	h30	S34	0.315	10	41
			h42	S47	0.574	8	
			h32	S36	0.630	4	
			h31	S35	0.722	6	

			h37/b1	S37/b1		13	
b.39.1	**L14**	0.489	H96	H3	0.130	4	24
			H71	E29	0.648	4	
			H92	G19	0.722	4	
			H61	E19	0.500	4	
			H95	H2	0.611	8	
d.41.4	**L16**	0.493	H89	G16	0.296	8	45
			H39	D15	0.370	14	
			H42	D18	0.630	8	
			H81	G7	0.667	4	
			H80	G6	0.704	5	
			H40	D16	0.907	6	
d.58.15	**S10**	0.493	h41a	S46	0.315	4	21
			h43	S48	0.481	3	
			h31	S35	0.722	6	
			h38	S42	0.870	8	
d.47.1	**L11**	0.502	H45	D20	0.407	14	18
			H44	D19	0.630	4	
d.55.1	**L22**	0.502	H73	G1	0.296	9	42
			H2	B1	0.389	10	
			H61	E19	0.500	4	
			H50	E6	0.519	7	
			H26	D1	0.556	4	
			H99	J1	0.722	2	
			H3	B2	0.778	4	
			H35	D10	0.944	2	
c.22.1	**L4**	0.507	H27	D2	0.167	10	69
			H46a	D22	0.407	24	
			H19	B19	0.556	10	
			H26	D1	0.556	6	
			H28	D3	0.556	7	
			H22	B21	0.796	12	
e.24.1	**L1**	0.516	H76	G4	0.037	4	4
d.59.1	**L30**	0.516	H41	D17	0.037	17	41
			H38	D14	0.037	14	
			H46	D21	0.556	7	
			H41	D16	0.907	3	
c.12.1	**L15**	0.525	H88	G15	0.426	16	60
			H37	D13	0.444	14	
			H12	B12	0.519	8	
			H11	B10	0.556	3	
			H36	D12	0.685	12	
			H29	D4	0.926	4	
			H12	B11	0.963	3	
d.12.1	**L23**	0.529	H51	E7	0.907	4	4
a.16.1	**S15**	0.534	h26	S29	0.204	2	29
			h22	S24	0.333	17	
			h20	S23	0.593	5	
			h23a	S26	1.000	5	
j.84.1	**L10**	N/A	H42	D18	0.630	8	8
