# Supplementary material for: Ribosomal History Reveals Origins of Modern Protein Synthesis
Source: PLoS One. 2012 Mar 12;7(3):e32776. doi: 10.1371/journal.pone.0032776 (PMC3299690; doi:10.1371/journal.pone.0032776)
Supplement: Table S5 — List of species from which sequences of both SSU and LSU rRNA were used for the reconstruction of the trees of rRNA helices shown in Figure 2 . (PDF) [file pone.0032776.s013.pdf]

Table S5. List of species from which sequences of both SSU and LSU rRNA were used for the reconstruction of the trees of rRNA helices shown in Figure 2. A total of 31 sequences from each superkingdom of life was chosen to avoid sampling bias. Due to the limited availability of LSU sequences, 2 archaeal sequences were used in duplicate to keep the number of taxa equal in superkingdoms.

**Archaea**	**Bacteria**	**Eukarya**
Acidianus brierleyi	*Agrobacterium tumefaciens*	*Acorus gramineus*
Acidianus infernus	*Alcaligenes faecalis*	*Aedes albopictus*
Aeropyrum pernix	*Aquifex aeolicus*	*Arabidopsis thaliana*
Aeropyrum pernix	*Bacillus subtilis*	*Caenorhabditis elegans*
Archaeoglobus fulgidus	*Bordetella pertussis*	*Candida albicans*
Desulfurococcus mobilis	*Borrelia burgdorferi*	*Citrus aurantium*
Haloarcula marismortui	*Bradyrhizobium japonicum*	*Crithidia fasciculata*
Haloarcula marismortui	*Campylobacter coli*	*Cryptosporidium parvum*
Halobacterium halobium	*Chlamydia pneumoniae*	*Dictyostelium discoideum*
Halobacterium marismortui	*Chlamydophila abortus*	*Drosophila melanogaster*
Halococcus morrhuae	*Chlorobium limicola*	*Entamoeba histolytica*
Haloferax mediterranei	*Clostridium botulinum*	*Euglena gracilis*
Methanobacterium thermoautotrophicum	*Coxiella burnetii*	*Gelidium americanum*
Methanococcus jannaschii A	*Escherichia coli G*	*Giardia ardeae*
Methanococcus jannaschii B	*Fibrobacter succinogenes*	*Gnetum urens*
Methanococcus vannielii	*Flavobacterium odoratum*	*Guillardia theta*
Methanospirillum hungatei	*Haemophilus influenzae D*	*Homo sapiens*
Methanospirillum sp.	*Helicobacter pylori B*	*Hyphochytrium catenoides*
Natronobacterium magadii	*Lactobacillus amylolyticus*	*Mucor racemosus*
Pyrobaculum islandicum	*Nannocystis exedens*	*Nosema apis*
Pyrococcus abyssi	*Neisseria gonorrhoeae*	*Onikusa pristoides*
Pyrococcus horikoshii	*Pirellula marina*	*Oryza sativa*
Stygiolobus azoricus	*Rickettsia prowazekii*	*Palmaria palmata*
Sulfolobus acidocaldarius	*Serpulina hyodysenteriae*	*Physarum polycephalum*
Sulfolobus shibatae	*Staphylococcus aureus*	*Plasmodium falciparum*
Sulfolobus solfataricus	*Stigmatella aurantiaca*	*Saccharomyces cerevisiae*
Thermococcus celer	*Synechocystis sp.*	*Styela plicata*
Thermofilum pendens	*Thermotoga maritima*	*Tetrahymena pyriformis*
Thermoplasma acidophilum	*Thermus thermophilus*	*Trepomonas agilis*
Pyrobaculum neutrophilum	*Treponema pallidum*	*Trypanosoma brucei*
Thermococcus acidaminovorans	*Vibrio cholerae*	*Xenopus laevis*
