# Supplementary material for: Ribosomal History Reveals Origins of Modern Protein Synthesis
Source: PLoS One. 2012 Mar 12;7(3):e32776. doi: 10.1371/journal.pone.0032776 (PMC3299690; doi:10.1371/journal.pone.0032776)
Supplement: Text S1 — Phylogenetic analysis of molecular structure. (DOC) [file pone.0032776.s014.doc]

**S1 Phylogenetic analysis of molecular structure**

We used molecular structure to generate intrinsically rooted trees that describe the evolution of structures in RNA molecules [1] or structures in entire protein repertoires [2]. These methods “embed structure and function directly into phylogenetic analysis”. The experimental strategy unifies phylogenetics and structural biology, mines real data robustly, and has been employed in a number of important applications. For example, it has been used to reconstruct deeply rooted phylogenies of the living world [1,3,4], uncover reductive evolutionary tendencies in proteomes and a cellular origin for the tripartite world [5], trace the origin and evolution of metabolic networks and proteins [6], study the evolution of metallomes [7], explore the origins of amino acid charging and the genetic code [8] understand the evolution of important functional RNAs including SINE RNAs [9], tRNA [10], 5S rRNA [11], and RNase P [12], and trace evolution of RNA structure in ribosomes [13].

Phylogenetic trees are explicit statements about the history of a biological system. These graph representations are branching diagrams with branches (nodes) and leaves (taxa). They are built from data, observable features (characters) that are characteristic of the system. Two kinds of trees are used in our studies, trees of rRNA structural elements (molecular substructures) and trees of domain structures (Figure 1). While these trees exploit the shared-and-derived tenet of cladistic analysis, they are atypical and some of their major properties (which have been discussed extensively elsewhere [2,3,10,14]) deserve elaboration.

1. *Trees are universal phylogenetic statements that are intrinsically rooted and are highly unbalanced.* Trees are derived from a comprehensive census of either substructures in rRNA molecules or protein domain 3D structures in genomes that have been completely sequenced. Phylogenies have branches that represent lines of direct descent and describe the natural history of rRNA or the natural history of protein domain structures that are known. Consequently, they are phylogenetic statements that apply to the entire world of rRNA and proteins. The model of character state transformation (evolutionary model) used in phylogenetic reconstructions (described in Methods) produces trees that are rooted. Establishing evolution’s arrow does not require of local external hypotheses of relationship (e.g., “outgroup” taxa). Moreover, trees are phylogenetic statements that are derived from molecules that are modern. Consequently, any claims from these trees are necessarily linked to the design and structure of extant molecules and not to those of their predecessors that were perhaps lost in evolution. An analysis of statistics of tree shape and symmetry (e.g., for trees of domain structures [14]) indicates trees are highly unbalanced and suggests that semi-punctuated evolutionary processes [15] are important drivers of structural discovery in rRNA and proteins. For example, N_bar and cherry count measures of imbalance (see Methods) for the combined LSU and SSU rRNA tree of Figure 1 were compared with those from 100 randomly generated trees or 100 trees generated by the uniform speciation (Yule) model. The N-bar value for the combined LSU and SSU tree (31) was greater than those expected in Yule trees (9) and random trees (21). Therefore, trees are highly asymmetric and do not fit a random branching model. Similarly, cherry counts for the combined LSU, SSU tree (36) fall outside the rejection limits rejecting both the random and uniform speciation (Yule) models at 0.05 significance levels. Yule model upper/lower rejection limits were 51/50 and random model upper/lower rejection limits were 39/38. Tree imbalance is expected; the total number of rRNA motifs is finite [16] and the number of protein folds will probably not exceed ~1,600 [17]. This suggests for example that the discovery of individual folds occurs at extraordinarily low rate (once every ~10^6^-10^7^ years; [18]) and that each speciation event that produces a new domain structure must be regarded as a unique and rare event (a ‘punctuation’) in evolutionary history. Similar arguments can be brought to explain the rarity of structural motifs in RNA.
2. *The leaves of the trees (taxa) are molecular structures.* In contrast with typical organism phylogenies used for example in systematic biology, the leaves of our trees are not organisms or molecules representing those organisms. Instead, leaves describe structural parts of molecules or molecular repertoires and the trees describe their evolution. These parts do not need to be specific or diagnostic to a lineage of an organism to describe historical relationships. Even parts that are shared by all lineages have a history that is made explicit in the trees. In trees of rRNA substructural elements, taxa are rRNA substructures of different kinds. We have generated trees of rRNA helices, hairpin loops, internal loops, and bulges, but in this study we focus on the history of rRNA helical segments. rRNA helices or regions with nucleotide stacking are the backbone of the rRNA structure and have been decomposed in molecules for evolutionary analysis. To ensure homology each kind of rRNA substructure was used to reconstruct separate trees. In trees of domain structures, taxa are domains defined at different levels of the SCOP structural hierarchy: class, fold, fold superfamily (FSF), and fold family [19,20]. Here we selected the FSF hierarchical level of domain structure as taxa, since this level coarse-grains the 3D atomic structure of proteins and is evolutionarily highly conserved. In this regard, phylogenomic analysis at the FSF level offers a higher level of certainty that proteins belonging to this hierarchical level share a common evolutionary origin [4], especially because families unified in an FSF show good structural and functional evidence of common ancestry [20]. Very much as with taxa of trees of rRNA substructures or ‘trees of life’ (i.e. trees of organismal species), each hierarchical level of domain structure is used separately in each tree reconstruction exercise to ensure homology. Note that when reconstructing trees of species the definition of what is a species is sometimes controversial (e.g. the definition of α−proteobacteria given pervasive horizontal gene transfer). Similarly, the reconstruction of trees of structures is also dependent on the validity and definition of terminal taxa. For example, a phylogenomic analysis of folds places trust on how SCOP assigns FSF to folds.
3. *The internal nodes of the trees define relative chronologies of structural diversification.* While the leaves of the trees correspond to rRNA structural components or protein domain structures, the nodes represent structural diversification events that occur as changes in features of rRNA structure or the popularity and spread of domain structures in proteomes develop in time. Consequently, nodes close to the base of the tree reflect more ancient events than those close to the leaves. Internal nodes in trees of rRNA describe the birth of substructures in the context of other ribosomal substructures [10,21]. Interestingly, the putative existence of hard polytomies depicting simultaneous divergence of substructures illustrates how rRNA duplications or other molecular rearrangements produce homologous segments in different parts of the molecule [21]. In this regard, absence of hard polytomies in the rRNA trees we have reconstructed (e.g., Figure 2) provides evidence of gradual buildup of rRNA substructures in the course of evolution. Internal nodes of the tree of domain structures describe the birth and structural diversification of domains, interpreted in the context of degenerate mappings (and associated neutral nets) that exist between the space of protein sequences and the space of proteins structures (reviewed in [18,22]). As protein sequences harboring a primordial FSF drift by mutation in sequence space, seek stability, diversify, and populate the proteome of a primordial organism, new sequences are discovered in the space of possible sequences that fold into new FSF structures. The rare finding of a new set of sequences with a novel attribute of protein structure constitutes a molecular speciation event and an internal node in the tree. Algorithmically and working down from the leaves to the root of the tree, a node that gives rise to two extant domains represents a hypothetical ancestor of these domains that, if it existed today, would in general have a greater genomic abundance than its children domains across the proteomes that are examined. Similarly, a hypothetical ancestor of an extant domain and of another hypothetical ancestor, or of two hypothetical ancestors, would in general have a greater abundance than its children. Since trees of rRNA substructures or domain structures are highly unbalanced and the timing of structural discovery is largely defined by molecular speciation, the number of internal nodes in lineages defines the relative age of domains (see Methods).
4. *The features (phylogenetic characters) that are used to build trees describe rRNA structure in molecular lineages or the abundance of domain structures in proteomes.* Characters used to build trees of rRNA substructures are features of structure associated with molecular lineages of rRNA molecules in individual organisms. Similarly, characters used to build trees of domain structure are the numbers of domain sequences that fold into domain structures in each proteome, i.e. the genomic abundance of domain structures. By definition, molecules in organisms and proteomes generally represent lineages that have distinct histories and fulfill the requirement of character independence that is necessary for phylogenetic analysis. We note however that crucial factors complicate phylogenetic analysis, including the effect of convergent evolutionary processes (i.e., those that lead independently to a similar outcome) and horizontal transfer [23]. While these processes have the potential of obliterating patterns of descent, their effect on structure appears limited [18]. Similarly, the existence of lineages arising from multiple ancestors could complicate phylogenetic interpretation [23]. However, almost all proteins share structural similarities with other proteins and are related by common ancestry [20] and phylogenetic and large-scale statistical analyses have shown that convergent evolution of domain structures is rare [24]. Consequently, the diversity of protein structures arose from a small set of ancestral molecules by descent with modification, with functions enhanced by domain rearrangement processes [14]. Moreover, a scenario of multiple origins is less parsimonious and unlikely in evolution of life, as shown recently for sequences [25].
5. *The criterion of primary homology rests on the feature of rRNA substructure being studied or genomic abundance levels of individual domains that exist in individual proteomes.* Homology can be defined as correspondence arising from common ancestry and its analysis represents a complex theoretical problem. The criterion of primary homology in our study defines general patterns of correspondence in geometrical features of rRNA substructures and genomic abundance of domain structures in proteomes using topology and ontogenetic criteria (both of which are linked) that follow a transformation sequence of ordered and polarized multistate characters. The criterion depends on topographic correspondence between substructures in rRNA and on how domains distribute and are reused in proteomes.

**References**

1. Caetano-Anolles G (2002) Evolved RNA secondary structure and the rooting of the universal tree of life. J Mol Evol 54: 333 - 345.

2. Caetano-Anolles G, Caetano-Anolles D (2003) An evolutionarily structured universe of protein architecture. Genome Res 13: 1563 - 1571.

3. Wang M, Caetano-Anollés G (2006) Global phylogeny determined by the combination of protein domains in proteomes. Mol Biol Evol 23: 2444-2454.

4. Yang S, Doolittle RF, Bourne PE (2005) Phylogeny determined by protein domain content. Proc Natl Acad Sci USA 102: 373-378.

5. Wang M, Yafremava LS, Caetano-Anollés D, Mittenthal JE, Caetano-Anollés G (2007) Reductive evolution of architectural repertoires in proteomes and the birth of the tripartite world. Genome Res 17: 1572-1585.

6. Caetano-Anollés G, Yafremava LS, Gee H, Caetano-Anollés D, Kim HS, et al. (2009) The origin and evolution of modern metabolism. Int J Biochem Cell Biol 41: 285-297.

7. Dupont CL, Butcher A, Valas RE, Bourne PE, Caetano-Anollés G (2010) History of biological metal utilization inferred through phylogenomic analysis of protein structures. Proc Natl Acad Sci USA 107: 10567-10572.

8. Sun FJ, Caetano-Anollés G (2008) Evolutionary patterns in the sequence and structure of transfer RNA: A window into early translation and the genetic code. PLoS ONE 3.

9. Sun FJ, Fleurdépine S, Bousquet-Antonelli C, Caetano-Anollés G, Deragon JM (2007) Common evolutionary trends for SINE RNA structures. Trends in Genet 23: 26-33.

10. Sun FJ, Caetano-Anollés G (2008) The origin and evolution of tRNA inferred from phylogenetic analysis of structure. J Mol Evol 66: 21-35.

11. Sun FJ, Caetano-Anollés G (2009) The evolutionary history of the structure of 5S ribosomal RNA. J Mol Evol 69: 430-443.

12. Sun F-J, Caetano-Anolles G (2010) The ancient history of the structure of ribonuclease P and the early origins of Archaea. BMC Bioinformatics 11: 153.

13. Caetano-Anolles G (2002) Tracing the evolution of RNA structure in ribosomes. Nucleic Acids Res 30: 2575 - 2587.

14. Wang M, Caetano-Anollés G (2009) The evolutionary mechanics of domain organization in proteomes and the rise of modularity in the protein world. Structure 17: 66-78.

15. Webster AJ, Payne RJH, Pagel M (2003) Molecular Phylogenies Link Rates of Evolution and Speciation. Science 301: 478-.

16. Leontis NB, Lescoute A, Westhof E (2006) The building blocks and motifs of RNA architecture. Curr Op Struct Biol 16: 279-287.

17. Levitt M (2007) Growth of novel protein structural data. Proc Natl Acad Sci USA 104: 3183-3188.

18. Caetano-Anollés G, Wang M, Caetano-Anollés D, Mittenthal JE (2009) The origin, evolution and structure of the protein world. Biochem J 417: 621-637.

19. Andreeva A, Howorth D, Chandonia JM, Brenner SE, Hubbard TJP, et al. (2008) Data growth and its impact on the SCOP database: New developments. Nucleic Acids Research 36: D419-D425.

20. Murzin AG, Brenner SE, Hubbard T, Chothia C (1995) SCOP: A structural classification of proteins database for the investigation of sequences and structures. J Mol Biol 247: 536-540.

21. Sun FJ, Caetano-Anollés G (2010) The Origin of modern 5s rRNA: A case of relating models of structural history to phylogenetic data. J Mol Evol 71: 3-5.

22. Caetano-Anollés G, Mittenthal J (2010) Exploring the interplay of stability and function in protein evolution: New methods further elucidate why protein stability is necessarily so tenuous and stability-increasing mutations compromise biological function. BioEssays 32: 655-658.

23. Wang M, Boca SM, Kalelkar R, Mittenthal JE, Caetano-Anollés G (2006) A phylogenomic reconstruction of the protein world based on a genomic census of protein fold architecture. Complexity 12: 27-40.

24. Gough J (2005) Convergent evolution of domain architectures (is rare). Bioinformatics 21: 1464-1471.

25. Theobald DL, Wuttke DS (2005) Divergent evolution within protein superfolds inferred from profile-based phylogenetics. J Mol Biol 354: 722-737.
