# Supplementary material for: Ribosomal History Reveals Origins of Modern Protein Synthesis
Source: PLoS One. 2012 Mar 12;7(3):e32776. doi: 10.1371/journal.pone.0032776 (PMC3299690; doi:10.1371/journal.pone.0032776)
Supplement: Text S2 — Evolution of the functional rRNA core. (PDF) [file pone.0032776.s015.pdf]

S2 Evolution of the functional rRNA core

The SSU rRNA consists of one molecule (16S/18S rRNA) that is divided into three major domains that fold independently into compact structures with 50 universal helices [1]. Phylogenetic trees show h44, the penultimate helical stem in the SSU rRNA, is the oldest (*nd* = 0) (Figure 2; Figure S1). This helix is one of the most functionally important ribosomal substructures (Table S1). It interacts with other SSU substructures responsible for mRNA decoding and with the LSU rRNA forming a functional relay [2]. Most of the interactions of the mRNA and the tRNA are centered in this helix. This relay is proposed to link processes in the SSU decoding site with LSU-based processes such as peptide bond formation and the release of elongation factors, thus modulating intersubunit interactions [2]. Helices h23, h24, h28, h30 and h34 are primordial (*nd* = 0.185-0.315); the first four define the A, P and E sites of the SSU and the last is involved in tRNA translocation during the elongation cycle of translation [3]. Some helices that are proximal to these ancient elements, such as helices h27, h29 and h31, are recent (*nd* = 0.444-0.722), suggesting they evolved after basic mechanisms were already established in the proto-ribosome, perhaps to refine established functions.

The LSU rRNA consists of 2-3 molecules (23S/28S and 5S/5.8S rRNA) that are about twice the size of SSU rRNA, are divided into six domains (I – VI) (5S rRNA is considered the seventh) and harbor 100 universal helices [2]. Trees show many functionally important regions of LSU rRNA are primordial (Figure 2; Figure S1). Helix H38 is one of the oldest substructures (*nd* = 0.037). It starts in the back of the particle, bends by about 90° and protrudes toward the SSU between domain V and 5S rRNA forming a crucial link between the two subunits [2]. Helices H73-H75, H89 and H90 that make up most of the catalytic core, the peptidyl transferase center (PTC) involved in peptide bond synthesis [4], are also relatively ancient (*nd* = 0.296). The helical regions form the base of the polypeptide exit tunnel. In addition helices H2 and H7 of domain I (*nd* = 0.389), helices H26, H35, H35a, and H40 of domain II (*nd* = 0.6-0.9), helix H52 of domain III (*nd* = 0.648), and helices H61, H64, and H65 of domain IV (*nd* = 0.5-0.7) which are derived compared to helices of domain V, also comprise the peptide exit tunnel. Helices, H32 and H69 that directly interact with the SSU are also derived (*nd* = 0.74). As with SSU, not all substructures that are proximal to the functional center are primordial or follow a serial chronology. Derived structural elements were therefore added to a basic functional proto-ribosomal unit later in evolution. This suggests the proto-ribosome was able to perform its function, perhaps less efficiently, with a simpler structure.

It is noteworthy that patterns of accretion of helices in LSU rRNA inferred from phylogenetic analysis of structure (our phylogenetic model [PM]) generally agree with accretion patterns inferred from A-minor acceptor-donor interactions (the A-minor interaction model [AM] of Bokov and Steinberg [5]). Figure S2 presents chronologies of structural accretion derived from both models. In both cases, segments of the PTC are among the oldest. However, PM reveals that regions of the LSU rRNA that are linked to ribosomal processivity are older than the PTC. The general match between the two models is remarkable, especially because AM assumptions provide grounds for conflict in any attempt of reconciliation. PM does not make assumptions about ribosomal origins but uses character argumentation criteria about the stability of molecules to inform about the evolutionary progression (see Text S1). In contrast, AM assumes that the ribosome originated in the PTC and that acceptor-helices that receive donor-adenosine stacks in A-minor motifs are ancient [5]. Thus, AM will place the PTC as the most ancient structure and will find the most parsimonious explanation of molecular accretion that is consistent with the appearance of acceptor helices prior to helices of donor stacks. If the PTC is not the most ancient substructure and the acceptor-donor assumption is violated, then AM accretion patterns may be incorrect and cannot be reconciled with phylogenetic data. We find PM validates the acceptor-donor assumption for most A-minor interactions (Figure 3F). Moreover, 50 out of 59 structural elements (85%) appear late in AM [5]. Consequently, most accretion patterns inferred from AM are not affected by the falsification of the PTC-first assumption of AM imposed by our model. AM and PM can be somehow reconciled.

Figure 4 shows the coordinated accumulation of helices in the SSU and LSU rRNA molecules derived from our timelines. The cumulative plots also reveal the very early appearance (*nd* < 0.3) of

substructures in the three SSU rRNA and six LSU rRNA domains. Remarkably, the initial accretion of helices occurred similarly in the two rRNA subunits, but only at *nd* > 0.3 the large subunit started to accumulate more helices than the small subunit.

The timeline of development of functional centers shows the early emergence of functionally important regions for ribosomal processivity, namely the A-site, P-site and E-site, tRNA interactions, and crucial intersubunit interactions (Figure 3D). Clustered around the h44 ratchet in 3D, SSU helices h11, h34, and h7 are responsible together with h44 for mRNA and tRNA translocation and are very ancient (*nd* = 0.06-0.13). The accretion of SSU helices harboring mRNA decoding, tRNA translocation and mRNA helicase activities (*nd* = 0.0-0.3) precede the origin of LSU substructures that make up the PTC, most of which appear together at *nd* ~ 0.3 (helices H73-H75, H89 and H90). Remarkably, the most ancient LSU helices H38, H41-42, H60, H67, and H96 (*nd* = 0.037-0.13), are all clustered around the more derived PTC in 3D (Figure 3), and the rapid and coordinated development of the PTC at *nd* ~ 0.3 agrees with the proposal that a duplication event [5,6] and a self-folding ribosomal module [7] was responsible for its formation.

The SSU rRNA interface harbors important inter-subunit bridges and tRNA contacts and consists of three primordial helices, h44, h23 and h24, and one derived helix, h14 (Figure 3B, Table S2). In contrast, the LSU rRNA interface is made up of derived helices H68, H70, H71, H69 and H64, and two primordial helices H67 and H62. These observations support the primordial nature of the processivity core and provide strong indication that the two ribosomal subunits evolved independently before they interacted in modern translation (see main text).

Figure 3E summarizes the start and end points of development of the core functions and uncovers the functional origins of the ribosome. In the core, h44, H38 and H67 together form more than half of inter-subunit bridge interactions. This is the processivity core of the ribosome (Figure 2). It performs the mechanically complex function of mRNA and tRNA binding and their translocation during the elongation cycle thus maintaining the reading frame and accuracy of translation. This is accomplished by the ratcheting action of the SSU relative to the LSU, which is driven by elongation factor G (EF-G) [8]. PTC functions are relatively simpler compared to ribosomal processivity. Both proximity and orientation of tRNA substrates in the PTC are the sole driving force during peptide bond synthesis [9-12]. The PTC is accessible to the tRNA only after selection by the decoding center mediated by elongations factor Tu (EF-Tu) [13]. Although the LSU can bind tRNAs by itself and synthesize peptide bonds with tRNA analogs [14] the rates of peptide bond synthesis are orders of magnitude lower. Peptide bond synthesis alone is not translation. Full length tRNAs are required to achieve reaction rates equal to that of the LSU-SSU complex and to maintain the conformation of the PTC [15]. Correct selection of tRNA induces a signaling cascade effecting structural changes characteristic of allosteric mechanisms. Since SSU-facilitated selection of the correct aminoacyl-tRNA is the rate-limiting step for PTC activity we contend that the processivity center of the ribosome evolved before others to facilitate template directed polymerization.

References

1. Moore PB, Steitz TA (2003) The structural basis of large ribosomal subunit function. Annual Review of Biochemistry 72: 813-850.
2. Cate JH, Yusupov MM, Yusupova GZ, Earnest TN, Noller HF (1999) X-ray crystal structures of 70S ribosome functional complexes. Science 285: 2095-2104.
3. Kubarenko A, Sergiev P, Wintermeyer W, Dontsova O, Rodnina MV (2006) Involvement of Helix 34 of 16 S rRNA in Decoding and Translocation on the Ribosome. J Biol Chem 281: 35235-35244.
4. Nissen P, Hansen J, Ban N, Moore PB, Steitz TA (2000) The Structural Basis of Ribosome Activity in Peptide Bond Synthesis. Science 289: 920-930.
5. Bokov K, Steinberg SV (2009) A hierarchical model for evolution of 23S ribosomal RNA. Nature 457: 977-980.
6. Agmon I, Bashan A, Yonath A (2006) On ribosome conservation and evolution. Israel J Ecol Evol 52: 359-374.
7. Smith TF, Lee JC, Gutell RR, Hartman H (2008) The origin and evolution of the ribosome. Biol Direct 3.

8. Frank J, Agrawal RK (2000) A ratchet-like inter-subunit reorganization of the ribosome during translocation. Nature 406: 318-322.
9. Gregory ST, Dahlberg AE (2004) Peptide bond formation is all about proximity. Nat Struct Mol Biol 11: 586-587.
10. Beringer M, Rodnina MV (2007) The Ribosomal Peptidyl Transferase. Mol Cell 26: 311-321.
11. Schroeder GK, Wolfenden R (2007) The rate enhancement produced by the ribosome: An improved model. Biochemistry 46: 4037-4044.
12. Wallin G, Åqvist J (2010) The transition state for peptide bond formation reveals the ribosome as a water trap. Proc Natl Acad Sci USA 107: 1888-1893.
13. Schmeing TM, Voorhees RM, Kelley AC, Gao Y-G, Murphy FV, IV, et al. (2009) The Crystal Structure of the Ribosome Bound to EF-Tu and Aminoacyl-tRNA. Science 326: 688-694.
14. Sardesai NY, Green R, Schimmel P (1999) Efficient 50S Ribosome-Catalyzed Peptide Bond Synthesis with an Aminoacyl Minihelix. Biochemistry 38: 12080-12088.
15. Wohlgemuth I, Beringer M, Rodnina MV (2006) Rapid peptide bond formation on isolated 50S ribosomal subunits. EMBO Rep 7: 699-703.
