# Supplementary material for: Ribosomal History Reveals Origins of Modern Protein Synthesis
Source: PLoS One. 2012 Mar 12;7(3):e32776. doi: 10.1371/journal.pone.0032776 (PMC3299690; doi:10.1371/journal.pone.0032776)
Supplement: Text S3 — Role of tRNA in ribosomal evolution. (PDF) [file pone.0032776.s016.pdf]

S3 Role of tRNA in ribosomal evolution

tRNAs have two structurally, and functionally independent halves with independent evolutionary origins [1,2]. The top half to which amino acids are charged contains the acceptor and the TΨC arms and is ancient, while the bottom half contains the anticodon and dihydrouridine arms of the molecule and is derived. The independent origins of the two functional halves of tRNA [1,2], their almost exclusive interaction with only one rRNA subunit, and the independent history of these interactions (Figure 3C), suggest rRNA subunits (originally perhaps with different functions) were recruited for translation only after a modern cloverleaf tRNA-like molecule evolved. tRNA-like structures involved in viral RNA replication [3], bacterial plasmid replication [4], and organellar DNA replication [5] that are proposed to be relics of primitive tRNAs [6] support our hypothesis. The acceptor arm is the most ancient part of the tRNA and the evolution of the tRNA structure suggests that the modern tRNA molecule had evolved much earlier than the modern ribosome [1,7]. Furthermore, minihelices corresponding to the top-half (acceptor arm) of tRNA can be substrates for ribosomal peptide synthesis [39] and EF-Tu [8]. However, while minihelices can mediate peptide bond formation the reaction rate is very slow in the absence of full sized tRNAs [9]. Thus we contend that modern translation evolved around tRNA by addition of new structural elements to a much simpler primitive ribosome. Mutational analyses of the newer rRNA and r-proteins structures do not abolish the functions but disrupt the efficiency of the translation process [10]. This corroborates the proposal that these structural components evolved to refine a less efficient system.

Most theories of ribosome evolution focus on peptidyl transfer and hence the PTC. However, the translation cycle consists of multiple processes preceding and followed by the peptidyl transferase reaction in initiation, elongation and termination of protein synthesis [11]. Elongation requires multiple, repetitive steps of tRNA selection and translocation involving conformational changes in the structure of LSU and SSU [12] and corresponding intersubunit movements with a ratchet-like rotation of the SSU relative to the LSU [13]. The transferase reaction in the PTC is the simplest of all of these processes, positioning the charged tRNAs in an optimal, proximal orientation with hardly any chemical contribution to enhance the rate of peptide bond synthesis [14,15]. Instead, the 3' CCA end of the acceptor arm is critical for catalysis [16], making tRNA not a mere adapter but an active chemical contributor to translation [17]. The 3' CCA end is central to, and intimately involved in all steps of translation, including initiation, elongation and termination [18]. It also contributes to fidelity at various steps of the elongation cycle [19] and is as critical to peptide release during termination as it is during peptide bond synthesis. Contacts with this acceptor arm appear after the major transition suggesting modern catalysis mediated by the 3' end of the acceptor arm occurred well after the establishment of the PTC (Figure 3). Thus, ribosomes and tRNAs coevolved, turning a simpler, error prone and sluggish primitive replication process into a complex translation process that is accurate and faster.

References

1. Sun FJ, Caetano-Anollés G (2008) The origin and evolution of tRNA inferred from phylogenetic analysis of structure. J Mol Evol 66: 21-35.
2. Maizels N, Weiner AM (1999) The genomic tag hypothesis: What molecular fossils tell us about the evolution of tRNA. The RNA World. Second Edition ed: Cold Spring Harbor Laboratory Press. pp. 79-112.
3. Giegé R, Frugier M, Rudinger J (1998) tRNA mimics. Curr Op Struct Biol 8: 286-293.
4. Wegrzyn G, Wegrzyn A (2008) Is tRNA only a translation factor or also a regulator of other processes? J Appl Genet 49: 115-122.
5. Yu C-H, Liao J-Y, Zhou H, Qu L-H (2008) The rat mitochondrial Ori L encodes a novel small RNA resembling an ancestral tRNA. Biochem Biophy Res Commun 372: 634-638.
6. Maizels N, Weiner A (1994) Phylogeny from function: Evidence from the molecular fossil record that tRNA originated in replication, not translation. Proc Natl Acad Sci USA 91: 6729 - 6734.
7. Sun FJ, Caetano-Anollés G (2008) Evolutionary patterns in the sequence and structure of transfer RNA: A window into early translation and the genetic code. PLoS ONE 3.

8. Rudinger J, Blechschmidt B, Ribeiro S, Sprinzl M (2002) Minimalist Aminoacylated RNAs as Efficient Substrates for Elongation Factor Tu. Biochemistry 33: 5682-5688.
9. Wohlgemuth I, Beringer M, Rodnina MV (2006) Rapid peptide bond formation on isolated 50S ribosomal subunits. EMBO Rep 7: 699-703.
10. Triman KL, Jeffery CH (2007) Mutational Analysis of the Ribosome. Adv. Genet: 58: 89-119.
11. Rodnina MV, Savelsbergh A, Katunin VI, Wintermeyer W (1997) Hydrolysis of GTP by elongation factor G drives tRNA movement on the ribosome. Nature 385: 37-41.
12. Laurberg M, Asahara H, Korostelev A, Zhu J, Trakhanov S, et al. (2008) Structural basis for translation termination on the 70S ribosome. Nature 454: 852-857.
13. Frank J, Agrawal RK (2000) A ratchet-like inter-subunit reorganization of the ribosome during translocation. Nature 406: 318-322.
14. Beringer M, Rodnina MV (2007) The Ribosomal Peptidyl Transferase. Mol Cell 26: 311-321.
15. Schroeder GK, Wolfenden R (2007) The rate enhancement produced by the ribosome: An improved model. Biochemistry 46: 4037-4044.
16. Weinger JS, Parnell KM, Dorner S, Green R, Strobel SA (2004) Substrate-assisted catalysis of peptide bond formation by the ribosome. Nat Struct Mol Biol 11: 1101-1106.
17. Woese CR (2001) Translation: in retrospect and prospect. RNA 7: 1055-1067.
18. Brosius J (2001) tRNAs in the spotlight during protein biosynthesis. Trends Biochem Sci 26: 653-656.
19. Zaher HS, Green R (2009) Quality control by the ribosome following peptide bond formation. Nature 457: 161-166.
