# Supplementary material for: Ribosomal History Reveals Origins of Modern Protein Synthesis
Source: PLoS One. 2012 Mar 12;7(3):e32776. doi: 10.1371/journal.pone.0032776 (PMC3299690; doi:10.1371/journal.pone.0032776)
Supplement: Text S4 — Origin and evolution of r-proteins. (PDF) [file pone.0032776.s017.pdf]

S4 Origin and evolution of r-proteins

Phylogenomic analysis of domain structure reveals important and consistent patterns of protein evolution (reviewed recently in Caetano-Anollés et al. [1]). The most ancient FSF domains are universally present in all organisms and with time they are first lost in primordial archaeal lineages and then in eukaryal and bacterial lineages [2-5]. In turn, the rather late gain of Bacteria-specific, and then, Eukarya-specific and Archaea-specific structures, signal the emergence of superkingdoms. These same patterns were also observed in the diversification of ancient RNA molecules such as tRNA, 5S rRNA and ribonuclease P RNA, with RNA substructures specific to Archaea appearing before substructures specific to other superkingdoms [6-9]. Collectively, these studies revealed the origin of the tripartite cellular world, highlighting three evolutionary epochs [2]: an ancient 'architectural diversification' period (Epoch 1) in which ancient molecules emerged and diversified and proteomes were highly homogeneous, a 'superkingdom specification' period (Epoch 2) in which molecules sorted in emerging organismal lineages, and a late 'organismal diversification' period (Epoch 3) in which molecular lineages diversified and became specific to superkingdoms and notable proteome expansions occurred in Eukarya. In this timeline, reduction of structural repertoires was mostly confined to primordial archaeal lineages at the end of Epoch 1, an observation that is also confirmed in the phylogenomic tree of Figure 5. Remarkably such reductive evolution patterns were also observed in r-proteins families [10].

A recent study of evolutionary mechanisms of domain organization and modularity in the protein world also revealed fundamental evolutionary patterns [3] that are relevant to this study. Early during Epoch 1 ($nd_P < 0.1$) multifunctional single domain proteins dominated, domains fused to form multi-domain proteins, and proteins with fewer functions started to evolve ($nd_P = 0.1$-0.3). Later on, new domain combinations massively emerged as a result of fusion and fission activities in a 'big bang' ($nd_P > 0.6$) that coincides with the rise of metazoa during the onset of Epoch 3. However, during $nd_P = 0.32$-0.40, the fusion of domains and discovery of protein FSFs notably ceased. This 'gap' could indicate a fundamental revision of the protein biosynthetic apparatus after which the rate of discovery of new FSF architectures increased drastically. If this drastic improvement could be attributed to a single event, this event would be the enhancement of protein synthesis efficiency by factor-mediated translation driven by GTP hydrolysis. EF-G catalyzed elongation increases protein synthesis more than 50 fold [11]. The GTPase activity of EF-G requires and is strongly stimulated by r-protein L7/L12 [12], which appears at $nd_P = 0.42$ in our timelines (Figure 4B). It is striking to note that this event corresponds to development of the GTPase associated center of the LSU rRNA and the corresponding age of the L7/L12 protein complex associated with it. The rapid protein diversification seen in the tree of FSFs that occurs in a very defined clade of the tree (Figure 5A) and the change in r-protein-rRNA age congruence (Figure 5B) can both be explained by the sharp increase in the overall processivity of the ribosome. We regard this as a second major transition in the evolution of the ribosome. We propose that during this revision process proteins refined the structure of the rRNA machinery and increased processivity. The new RNP apparatus was much more efficient than its predecessor. Many experiments that truncate or delete r-proteins resulting in decreased activity of the ribosome confirm our hypothesis [13,14].

References

1. Caetano-Anollés G, Wang M, Caetano-Anollés D, Mittenthal JE (2009) The origin, evolution and structure of the protein world. Biochem J 417: 621-637.
2. Wang M, Yafremava LS, Caetano-Anollés D, Mittenthal JE, Caetano-Anollés G (2007) Reductive evolution of architectural repertoires in proteomes and the birth of the tripartite world. Genome Res 17: 1572-1585.
3. Wang M, Caetano-Anollés G (2009) The evolutionary mechanics of domain organization in proteomes and the rise of modularity in the protein world. Structure 17: 66-78.
4. Wang M, Boca SM, Kalelkar R, Mittenthal JE, Caetano-Anollés G (2006) A phylogenomic reconstruction of the protein world based on a genomic census of protein fold architecture. Complexity 12: 27-40.
5. Kim KM, Caetano-Anollés G (2012) The evolutionary history of protein fold families and proteomes confirms that the archaeal ancestor is more ancient than the ancestors of other superkingdoms. BMC Evol Biol 12: 13.

6. Sun FJ, Caetano-Anollés G (2008) The origin and evolution of tRNA inferred from phylogenetic analysis of structure. J Mol Evol 66: 21-35.
7. Sun FJ, Caetano-Anollés G (2009) The evolutionary history of the structure of 5S ribosomal RNA. J Mol Evol 69: 430-443.
8. Sun F-J, Caetano-Anolles G (2010) The ancient history of the structure of ribonuclease P and the early origins of Archaea. BMC Bioinformatics 11: 153.
9. Sun FJ, Caetano-Anollés G (2008) Evolutionary patterns in the sequence and structure of transfer RNA: Early origins of Archaea and viruses. PLoS Comp Biol 4: e1000018
10. Lecompte O, Ripp R, Thierry J-C, Moras D, Poch O (2002) Comparative analysis of ribosomal proteins in complete genomes: an example of reductive evolution at the domain scale. Nucl Acids Res 30: 5382-5390.
11. Rodnina MV, Savelsbergh A, Katunin VI, Wintermeyer W (1997) Hydrolysis of GTP by elongation factor G drives tRNA movement on the ribosome. Nature 385: 37-41.
12. Savelsbergh A, Mohr D, Wilden B, Wintermeyer W, Rodnina MV (2000) Stimulation of the GTPase activity of translation elongation factor G by ribosomal protein L7/12. J Biol Chem 275: 890-894.
13. Maguire BA, Beniaminov AD, Ramu H, Mankin AS, Zimmermann RA (2005) A protein component at the heart of an rna machine: The importance of protein L27 for the function of the bacterial ribosome. Mol Cell 20: 427-435.
14. Takyar S, Hickerson RP, Noller HF (2005) mRNA helicase activity of the ribosome. Cell 120: 49-58.
