# Supplementary material for: Ribosomal History Reveals Origins of Modern Protein Synthesis
Source: PLoS One. 2012 Mar 12;7(3):e32776. doi: 10.1371/journal.pone.0032776 (PMC3299690; doi:10.1371/journal.pone.0032776)
Supplement: Text S5 — Assessing structural similarity to detect functional shifts. (PDF) [file pone.0032776.s018.pdf]

S5 Assessing structural similarity to detect functional shifts.

Apart from the ribosome there are no examples of natural RNP polymerases. Despite the absence of such a link, proto-ribosomes have been proposed to be part of primitive replication machinery [1-5]. Particularly interesting are models that couple origin and coevolution of the genetic code and RNA replication facilitated by proto-ribosomes [6-8]. Ribosomes and DNA/RNA polymerases use similar strategies of minor-groove recognition to maintain fidelity, and carry on functions that are 'processive' in nature, i.e. *that associate mechanical and biosynthetic molecular processes*. Fidelity permits an error-prone primitive self-replicating system to evolve into a complex system [9]. Interestingly, fidelity and processivity are tightly linked in ribosomes [10]. The absence of natural RNP replication enzymes represents a gap in evolutionary continuity and precludes the possibility of obtaining a natural phylogeny of RNP and protein polymerases. However, *in vitro* selected ribozymes substitute as dopplegängers for supposedly extinct molecules and provide means to test the likelihood of their existence [11]. Recent crystal structures of ribozymes involved in ligation and polymerization of RNA have helped understand the reaction mechanisms. Moreover, both natural and artificial functional RNAs share universal evolved sequence features that inherently define conformational order [12]. These features arise fundamentally from intrinsic properties dictating RNA self-organization [13,14]. Hence detection of any remote homology between rRNA substructures and replicase/polymerase dopplegängers, detected for example using pairwise structural alignments, would support the replicative role of proto-ribosomes. Our results show there are in fact structural similarities between substructures of *in vitro* engineered L1 RNA ligase and RNA polymerase ribozymes that are functional and ancient rRNA substructures making functional centers (Figure 6). The functional ribozymic substructures that are homologous involve stem A of the L1 RNA ligase and the P1-P2, P4-P5, and P6-P7 helical regions of the RNA polymerase (Figure 6A), substructures that hold the catalytic sites. Over half (54%) of homologous rRNA substructures appeared before the major transition at *nd* ~ 0.3, and all appeared before *nd* ~ 0.65 (Figure 6C). A substantial fraction of rRNA helices that are hit (44%) are crucial functional components of the ribosome and several of the other helices have important structural supportive roles, such as h11 (*nd* = 0.06) of the ancient SSU rRNA core, which interacts with S17. Six out of the seven most ancient are associated with fundamental processivity functions. Examples of primordial rRNA substructures that were repeatedly found to be homologous included the very ancient h34, h44, H38, and H76 crucial for mRNA helicase activity and decoding and for tRNA translocation, the H41-H42 coaxial stem supporting the GTPase center, and H67 supporting the crucial and ancient B2 bridge. Similarly, helices that make up the PTC (H73, H90, and H94) are all repeated homologs of the ligase and replicase substructures. Most remarkably, almost half of the 38 universal rRNA helices that have clear functional roles (Table S1) were homologous to functional substructures of the ligase/polymerase ribozymes, suggesting a clear link between extant and ancient replicative functions. We note however and caution that homology is not phylogeny and that any structural similarity between *in vitro* engineered ribozymes and an ancient rRNA scaffold does not show unambiguously that ancient replicative functions were recruited for protein synthesis. Any putative link must be supported by congruence with other sources of evidence. In our study, remote homology is linked to biases in the age and function of rRNA substructures derived from phylogenetic analysis of molecular structure (this study), biochemistry, and structural biology. For example, recent biochemical evidence linked to the PTC suggests that the most conserved of the two layers of unpaired bases of the catalytic center are proximal to relevant tRNA moieties and are required for peptidyl-tRNA hydrolysis (a processive function) but not for the more facile peptide bond synthesis reaction (a biosynthetic function) [15]. Collectively, all of these observations support the crucial evolutionary role of ribosomal processivity.

Results

1. Poole AM, Jeffares DC, Penny D (1998) The Path from the RNA World. J Mol Evol 46: 1-17.
2. Campbell JH (1991) An RNA replisome as the ancestor of the ribosome. J Mol Evol 32: 3-5.

3. Penny D (2005) An interpretive review of the origin of life research. Biol Philosophy 20: 633-671.
4. Taylor WR (2006) A molecular model for the origin of protein translation in an RNA world. J Theor Biol 243: 393-406.
5. Yakhnin AV (2007) A model for the origin of protein synthesis as coreplicational scanning of nascent RNA. Orig Life Evol Biosp 37: 523-536.
6. Bedian V (1982) The possible role of assignment catalysts in the origin of the genetic code. Orig Life 12: 181-204.
7. Mackinlay AG (1982) Polynucleotide replication coupled to protein synthesis: A possible mechanism for the origin of life. Orig Life 12: 55-69.
8. Stevenson DS (2002) Co-evolution of the genetic code and ribozyme replication. J Theor Biol 217: 235-253.
67. Ramakrishnan V (2009) The Ribosome: Some Hard Facts about Its Structure and Hot Air about Its Evolution. Cold Spring Harb Symp Quant Biol 2: 2.
68. Dong H, Kurland CG (1995) Ribosome mutants with altered accuracy translate with reduced processivity. J Mol Biol 248: 551-561.
69. Ellington AD, Chen X, Robertson M, Syrett A (2009) Evolutionary origins and directed evolution of RNA. International Journal of Biochemistry and Cell Biology 41: 254-265.
70. Kennedy R, Lladser ME, Wu Z, Zhang C, Yarus M, et al. (2010) Natural and artificial RNAs occupy the same restricted region of sequence space. RNA 16: 280-289.
71. Schultes EA, Hraber PT, LaBean TH (1999) Estimating the contributions of selection and self-organization in RNA secondary structure. J Mol Evol 49: 76-83.
72. Smit S, Yarus M, Knight R (2006) Natural selection is not required to explain universal compositional patterns in rRNA secondary structure categories. RNA 12: 1-14.
73. Youngman EM, Brunelle JL, Kochaniak AB, Green R (2004) The active site of the ribosome is composed of two layers of conserved nucleotides with distinct roles in peptide bond formation and peptide release. Cell 117: 589-599.
74. Gao H, Sengupta J, Korostelev A, Eswar N, Stagg Sm, Van Roey P, Agrawal RK, Harvey SC, Sali A, Chapman MS, Frank J (2003) Study of the structural dynamics of the *E. coli* 70S ribosome using real-space refinement. Cell 113**:** 789-801.
75. Kietrys AM, Szopa A, Basskowsk-Zywicka K (2009) Structure and function of intersubunit bridges in procaryotic ribosome. Biotechnologia 1:48-58.
